# Supplementary material for: A robotic prebiotic chemist probes long term reactions of complexifying mixtures
Source: Nat Commun. 2021 Jun 10;12:3547. doi: 10.1038/s41467-021-23828-z (PMC8192940; doi:10.1038/s41467-021-23828-z)
Supplement: Supplementary file 1 — Supplementary Information [file 41467_2021_23828_MOESM1_ESM.pdf]

Supplementary Information for:

A robotic prebiotic chemist probes long term reactions of complexifying mixtures

Silke Asche[1], Geoffrey J. T. Cooper[1], Graham Keenan[1], Cole Mathis[1] and Leroy Cronin[1]*

[1]*School of Chemistry, University of Glasgow, Joseph Black Building, University Avenue, Glasgow G12 8QQ, U.K.*

*Corresponding author email: lee.cronin@glasgow.ac.uk

Contents

A robotic prebiotic chemist probes long term reactions of complexifying mixtures 1

1 Hardware 3

2 Experimental Details 5

2.1 Dilution vs. product persistence 5

2.2 Chemical selection process 5

2.3 Mineral leaching 6

2.4 List of experiments 8

2.5 Instrumentation 10

2.5.1 Electrospray- Ionisation Mass Spectrometry (ESI-MS) 10

3 Mass Index calculation 11

3.1 Mass Index calculation 11

3.2 Mass Index trends 11

W .... **Error! Bookmark not defined.**

3.3 Mass Index calculation examples 12

4 Example experimental data 13

4.1 General 13

4.1.1 Input compositions used 13

4.1.2 pH measurements 14

4.1.3 Mineral controls 15

4.1.4 HPLC-DAD data 17

4.1.5 Online HPLC-MS 19

4.1.6 Offline HPLC-ESI-MS 19

4.2 Formula identification 23

4.2.1 Experimentally found values 23

4.2.2 Matching experimental and theoretical formulas 29

4.2.3 Product persistence over dilution 30
5 Decision Making Algorithm 31
5.1 Architecture 31
5.2 Measure of complexity 32
5.3 Decision making algorithm example 33
5.4 Alternative algorithms tested 34
5.4.1 Adaptive Mass Index 34
5.4.2 Weight by intensity index 38
5.4.3 Information entropy value 40
6 References 43

1 Hardware

The automated platform system consists of four main elements: the input system, the reactor, the in-line sample system and the sample storage. All elements are run by a modular python code written in-house. As a schematic of the whole build up is shown in the manuscript as Figure 2, so here we only elaborate on specific elements of the setup.

The input system consists of three Tricontinent syringe pumps each connected to a Tricontinent rotary 6-way distribution valve. Every valve is connected to six starting material bottles, resulting in a library of 18 input choices. Liquid is transported through 1/8 (3.2 mm) PTFE tubing with PEEK fittings.

The reactor, is a 100 mL round bottom flask with a specifically tailored head allowing attachment of screw fittings for the tubing connections and a reflux condenser, which is kept under a positive pressure of nitrogen gas, controlled by a flow controller (model 0254 by Brooks Instrument), ensuring a controlled reaction atmosphere. The temperature is controlled via an IKA RET ‘control-visc’ hotplate with a USB interface.

For the in-line analytical system, shown in Supplementary Figure **1** below, an HPLC-DAD (diode array detector) system is coupled to an Advion L-series benchtop mass spectrometer.

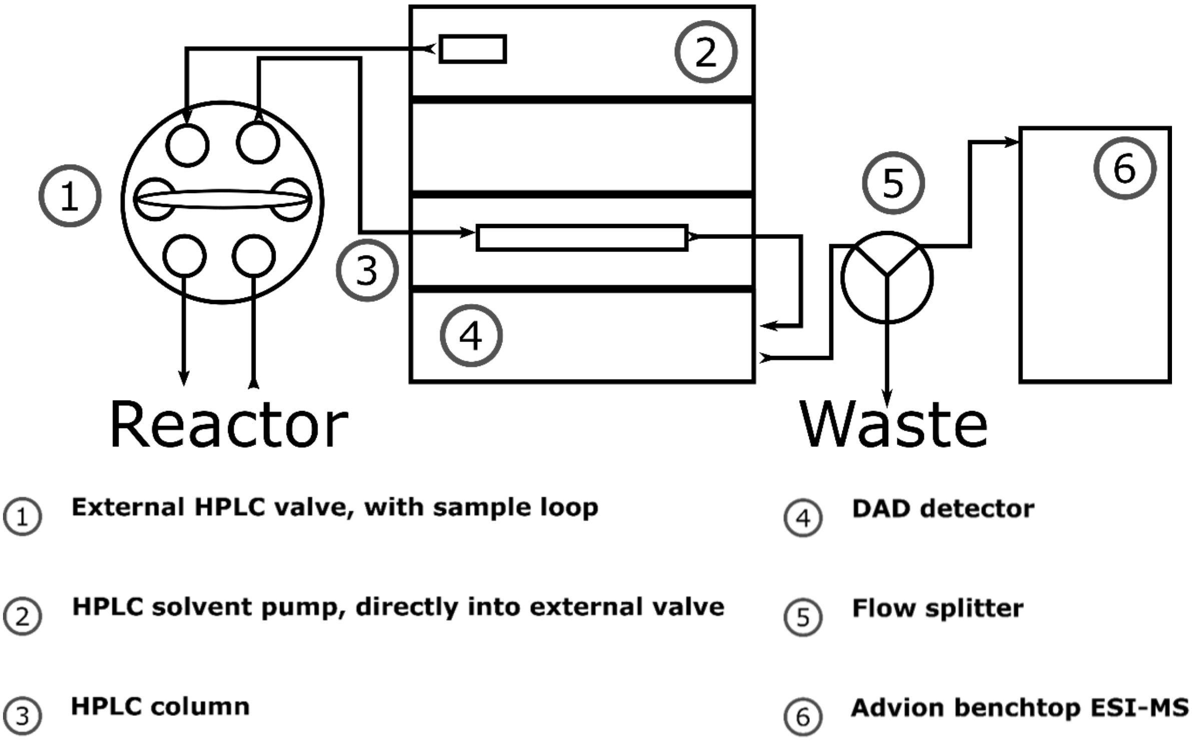

Supplementary Figure 1: In-line sampling and analytical system with external sample loop on the left, HPLC-DAD in the middle followed by a split valve, which injects into the ESI-MS on the right

The sample is drawn from the reactor by a peristaltic pump (Vapourtec model SF-10) and filtered through two 0.2 µm size nylon syringe filters fitted between a pair of Luer (Male) and Flat Bottom (Female) ETFE/Polypropylene adapters. It is injected into a 16 cm sample loop on an external, directly controlled, HPLC six-port switching valve. The sampling is triggered through the python code and actuated through an Arduino Mega 2560/RAMPs combination. Once the loop is loaded with fresh sample, the external sample valve is switched to flush the loaded sample loop with mobile phase from the LC-system and deliver the sample directly on the column. After the column, the sample moves through the diode array detector (DAD) and then into a split valve, which reduces the flow from 0.5 mL/min to 0.2 ml/min (the excess going to waste) ready for direct injection into the electrospray ionisation mass spectrometer (ESI-MS). This is triggered through a contact closure from the HPLC system, ensuring no delay in the parallel run time of both instruments.

The sample storage system (Supplementary Figure 2), which allows the storage of up to 20 samples of 50mL each in plastic sampling tubes, is build around an in-house designed 3D printed wheel. This wheel is based in a Geneva drive mechanism motion, allowing step-by-step increment of positions with high accuracy. The box in which the wheel system is contained is built from custom cut V-Slot aluminium rails. The column to hold the driven wheel, the drive wheel that increments the driven wheel, the stepper motor securing element, the supporting levelling arches and the solution dispensing part are all 3D printed on a Connex 500 printer with the translucent material RGD720. The base plate and the vial plate for holding the 50 ml plastic sample tubes is laser cut from acrylic plate. The levelling arches are all suited with an 8 mm ball bearing, and the Nema11 stepper motor that increments the drive is controlled through an Arduino MEGA.

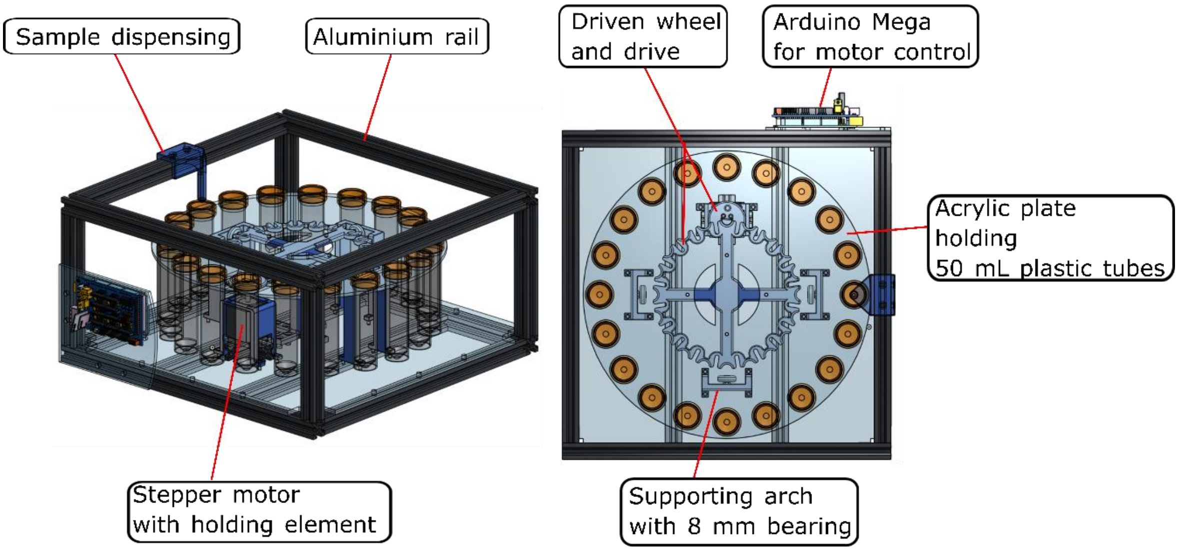

Supplementary Figure 2: Custom build sample storage system providing space for up to 20 samples of a volume of up to 50 mL per sample, which are collected in plastic centrifuge tubes. The lids of the tubes (orange), sit on the acrylic plate and are holding the vials in place. The 3d printed parts are shown in blue.

2 Experimental Details

2.1 Dilution vs. product persistence

In this study we are interested in the persistence of products, not of input reagents. If the experimental concept were linear and solely be based on periodically changing input compounds, every product would become diluted over time. However, our experimental concept is different, and with the inclusion of minerals, the reaction system is not linear. Over time, many of the components of the mixture will be diluted to infinitesimally low concentrations, but not all. Binding to mineral particles can lead to product build up on their surface which may then lead to species amplification, as well as a change of the species concentration in the reactor over time. Products in our system can be made from multiple reactions / input compositions, including the breakdown components of other product species, which can lead to some specific product species becoming prevalent, even under different input conditions. The critical question is therefore not about what will happen to the reagents upon serial dilution, but rather what products are formed and whether those products are formed robustly from the reaction mixture or only marginally. Those that are formed robustly (e.g. from multiple sets of reagent combinations) will persist (be amplified) in the face of dilution. When specific products persist through many reaction cycle, we say they have been amplified by the reaction, while all species that do not increase will be diluted out.

2.2 Chemical selection process

The starting material library was mostly chosen by purposefully avoiding specific chemical groups, meaning no “common” autocatalytic cycle precursors, sugars or amino acids have been selected. The aim was to solely concentrate on small functional building block molecules with no immediate connection or function. The following table shows the list of selected building blocks and some potential properties.

Supplementary Table 1: List of starting material reagents and their properties

Input chemical	Properties
Resorcinol	• weak organic acid<br>• can polymerize in condensation reactions
Pyruvic acid	• can be used to make glucose<br>• polymerizes and decomposes in touch with air
Acrylic acid	• polymerizes exposed to heat
Glycidol (2,3-epoxy-1-propanol)	• intermediate in the synthesis of glycerol, glycidyl ether, amines<br>• contact with earth metals may cause polymerization
Glycerol	• intermediate in carbohydrate and lipid metabolism
Carbonyldiimidazole	• enzyme cross linking agent<br>• coupling agent for amino acids and peptide synthesis
Ethyl acetate	• carboxylic acid ester<br>• exists in eukaryotes
Pyridine	• coordination chemistry ligand<br>• strong basic compound

Oxalic acid	• metabolite<br>• moderately acidic
Formamide	• monocarboxylic acid amide<br>• nucleic acid building block
Catechol	• weak organic acid<br>• participant in metabolic pathways
Ruthenium-(III)-chloride hydrate	• metal salt<br>• catalytic effects
Formaldehyde	• sugar precursor<br>• Miller-Urey intermediate
Copper-(II)-sulfate pentahydrate	• metal salt
Sulfuric acid	• strong acid<br>• corrosive
Nitric acid	• strong acid<br>• corrosive
Ammonium thiosulfate	• ammonium source<br>• sulphur part of earth crust
Potassium pyrophosphate	• potassium part of earth crust<br>• phosphate source (food)

2.3 Mineral leaching

Details about the mineral preparation and wash procedure can be found in the manuscript. To look into the effect or minerals leaching out into solution, a preliminary experiment was analysed by ICP-OES, testing if the elements present in the mineral, can be found in solution.

The ICP-OES (Inductively Coupled Plasma – Optical Emission Spectroscopy) analysis was performed on an Agilent 5100. Multi-standard solutions have been used for calibration. The samples have been treated with a 2% HNO_3 solution.

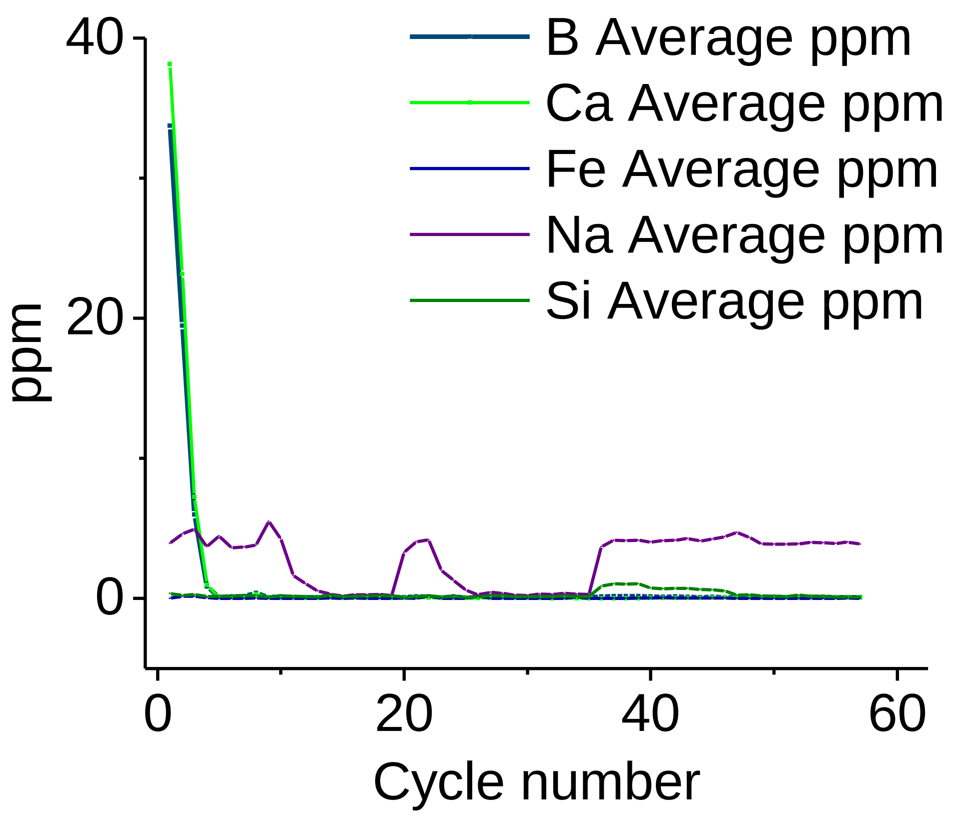

Supplementary Figure 3: ICP-OES plotted against the number of cycles. Boron, calcium, iron, sodium and silicon have been tested. The increase of sodium is based on input solution addition containing in the specific cycles.

The graph above shows, that it is possible for elements like calcium and boron to leach out of ulexite. This effect appears to be only present in the first 5 cycles, after which the concentration of these elements in solution drops to negligent amounts.

2.4 List of experiments

Supplementary Table 2: List of executed runs, their cycle number and their input solution in order of addition to the reactor. The run description specifically state the label used for the run in the SI, as well as figure 4 it and 5 of the main manuscript as labels have been changed for consistency and clarity.

RUN DESCRIPTION	CYCLE NUMBER	INPUT 1	INPUT 2	INPUT 3
A IN SI	1 to 17	resorcinol	pyruvic acid	ammonium thiosulfate
	18 to 26	copper-(II)-sulfate pentahydrate	oxalic acid	nitric acid
	27 to 36	copper-(II)-sulfate pentahydrate	oxalic acid	carbonyldiimidazole
	37 to 47	pyridine	ruthenium-(III)-chloride hydrate	ammonium thiosulfate
	48 to 59	pyruvic acid	resorcinol	catechol
	60 to 78	ammonium thiosulfate	carbonyldiimidazole	formamide
	79 to 88	pyruvic acid	formaldehyde	glycidol
	89 to 98	formaldehyde	acrylic acid	sulfuric acid
	99 to 110	carbonyldimidazole	pyridine	ruthenium-(III)-chloride hydrate
	111 to 113	ruthenium-(III)-chloride hydrate	sulfuric acid	ethyl acetate
B IN SI<br>A IN FIGURE 4<br>10 IN FIGURE 5	1 to 17	acrylic acid	potassium pyrophosphate	carbonyldiimidazole
	18 to 42	ethyl acetate	formamide	formaldehyde
	43 to 52	ruthenium-(III)-chloride hydrate	pyridine	copper-(II)-sulfate pentahydrate
	53 to 62	resorcinol	pyridine	formamide
	63 to 67	potassium pyrophosphate	ethyl acetate	formaldehyde
C IN SI<br>11 IN FIGURE 5<br>REPEAT OF RUN B	1 to 17	acrylic acid	potassium pyrophosphate	carbonyldiimidazole
	18 to 42	ethyl acetate	formamide	formaldehyde
	43 to 52	ruthenium-(III)-chloride hydrate	pyridine	copper-(II)-sulfate pentahydrate
	53 to 62	resorcinol	pyridine	formamide
	63 to 67	potassium pyrophosphate	ethyl acetate	formaldehyde

D IN SI<br>B IN FIGURE 4<br>12 IN FIGURE 5<br>REPEAT OF RUN B	1 to 17	acrylic acid	potassium pyrophosphate	carbonyldiimidazole
	18 to 42	ethyl acetate	formamide	formaldehyde
	43 to 52	ruthenium-(III)-chloride hydrate	pyridine	copper-(II)-sulfate pentahydrate
	53 to 62	resorcinol	pyridine	formamide
	63 to 67	potassium pyrophosphate	ethyl acetate	formaldehyde
E IN SI<br>C IN FIGURE 4	1 to 13	glycidol	pyruvic acid	sulfuric acid
	14 to 20	glycidol	resorcinol	catechol
	21 to 30	oxalic acid	sulfuric acid	acrylic acid
	31 to 43	sulfuric acid	potassium pyrophosphate	acrylic acid
	44 to 52	oxalic acid	formaldehyde	sulfuric acid
	53 to 70	acrylic acid	pyridine	sulfuric acid
	71 to 80	ammonium thiosulfate	oxalic acid	sulfuric acid
	81 to 92	ruthenium-(III)-chloride hydrate	carbonyldiimidazole	formamide
	93 to 100	ruthenium-(III)-chloride hydrate	potassium pyrophosphate	ethyl acetate
	101 to 112	nitric acid	oxalic acid	sulfuric acid
	113 to 125	ruthenium-(III)-chloride hydrate	potassium pyrophosphate	ruthenium-(III)-chloride hydrate
	126 to 129	ruthenium-(III)-chloride hydrate	potassium pyrophosphate	catechol
	130 to 160	ruthenium-(III)-chloride hydrate	potassium pyrophosphate	resorcinol
F IN SI<br>D IN FIGURE 4	1 to 1	pyruvic acid	ethyl acetate	oxalic acid
	13 to 20	acrylic acid	copper-(II)-sulfate pentahydrate	sulfuric acid
	21 to 43	glycidol	carbonyldiimidazole	oxalic acid
	44 to 56	ethyl acetate	pyruvic acid	ethyl acetate
	57 to 71	copper-(II)-sulfate pentahydrate	acrylic acid	glycidol
G IN SI<br>E IN FIGURE 4	1 to 10	oxalic acid	pyridine	catechol
	11 to 21	catechol	nitric acid	pyruvic acid

	22 to 34	glycerol	ammonium thiosulfate	formamide
	35 to 44	acrylic acid	potassium pyrophosphate	glycidol
	45 to 56	copper-(II)-sulfate pentahydrate	sulfuric acid	ammonium thiosulfate
	57 to 67	glycerol	ammonium thiosulfate	catechol
	68 to 76	glycidol	pyridine	pyruvic acid
H IN SI F IN FIGURE 4	1 to 12	glycidol	ammonium thiosulfate	carbonyldimidazole
	13 to 22	glycidol	formaldehyde	glycidol
	23 to 36	ammonium thiosulfate	copper-(II)-sulfate pentahydrate	catechol
	37 to 65	sulfuric acid	resorcinol	pyruvic acid
	66 to 80	sulfuric acid	ruthenium-(III)-chloride hydrate	carbonyldiimidazole
	81 to 90	oxalic acid	glycerol	resorcinol
	91 to 96	potassium pyrophosphate	catechol	resorcinol

2.5 Instrumentation

2.5.1 Electrospray- Ionisation Mass Spectrometry (ESI-MS)

The Bruker Maxis was used for offline analysis. Data is shown in the SI in Figure 11 to 16. The sample was run through a DIONEX Ultimate 3000 series HPLC-DAD set up with a RS (rapid separation) pump. Injected from the RS autosampler (WPS-3000 (T) RS) on an Agilent Infinity Lab Poroshell 120 Eclipse EC-C18 UHPLC 150 mm column, kept in a column compartment (TCC-3000SD) with a controlled temperature of 30 °C. The method used was a gradient method with 0.1 % formic acid added to LC-MS grade water and 0.1 % formic acid added to LC-MS grade acetonitrile (MeCN). The run started with 100 % aqueous phase. Over 4 minutes, the organic (MeCN) flow was increased to 10 %, after another 12 minutes it was at 7 0% MeCN. After 19 minutes runtime, a flow of 100 % organic mobile phase was reached. After that, the mobile phase was switched back to the initial 100 % water. The flow rate through the whole run was 0.7 mL/min and the total runtime was 26 minutes. The HPLC was connected to a Bruker MaXis Impact quadrupole time-of-flight mass spectrometer with an electrospray source, operating exclusively in positive mode. The instrument was calibrated with a sodium formate standard solution before each run. Samples were introduced into the MS at a dry gas temperature of 220 °C. The ion polarity for all MS scans recorded was positive, with the voltage of the capillary tip set at 4800 V, end plate offset at − 500 V, funnel 1 RF at 400 Vpp, funnel 2 RF at 400 Vpp, hexapole RF at 100 Vpp, ion energy 5.0 eV, collision energy at 5 eV, collision cell RF at 200 Vpp, transfer time at 100.0 μs, and

the pre-pulse storage time at 3.0 µs. The mass range was set to 50 – 2000 m/z. Data was analysed using the Bruker DataAnalysis v4.1 software suite.

3 Mass Index calculation

3.1 Mass Index calculation

The Mass Index is calculated as shown in Supplementary Figure 4. Additional example cases of specific cycles are shown below in**Error! Reference source not found.** to 7.

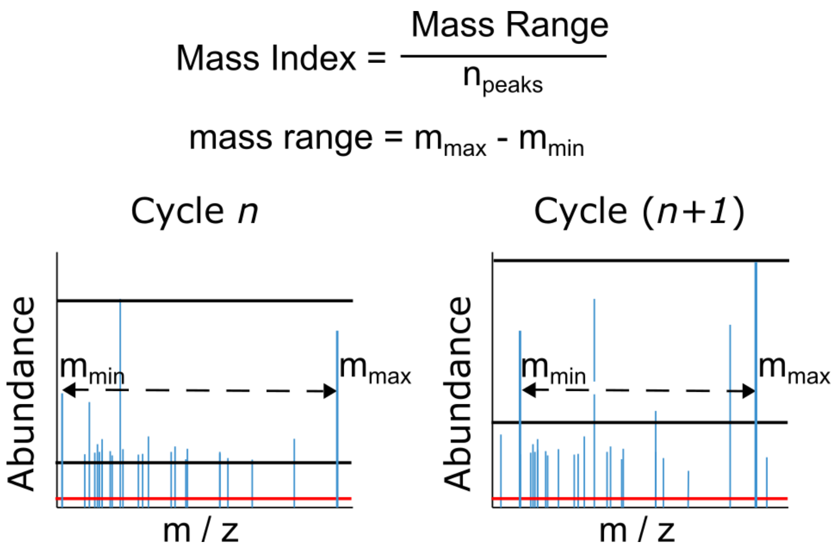

Supplementary Figure 4: Figure from Doran's paper(*1*), showing the Mass Index calculation. After thresholding(10^6 intensity), the heaviest and lightest peak are subtracted from each other to determine the mass range and divided through the number of peaks over threshold.

3.2 Mass Index trends

This table summarizes the expected behavior of the Mass Index in various cases.

Supplementary Table 3: Table depicting mass index trends

	Largest Product Constant	Number Product Species Constant
Mass Index UP	Fewer Product Species	Largest Product Increased
Mass Index DOWN	More Product Species	Largest Product decreased

3.3 Mass Index calculation examples

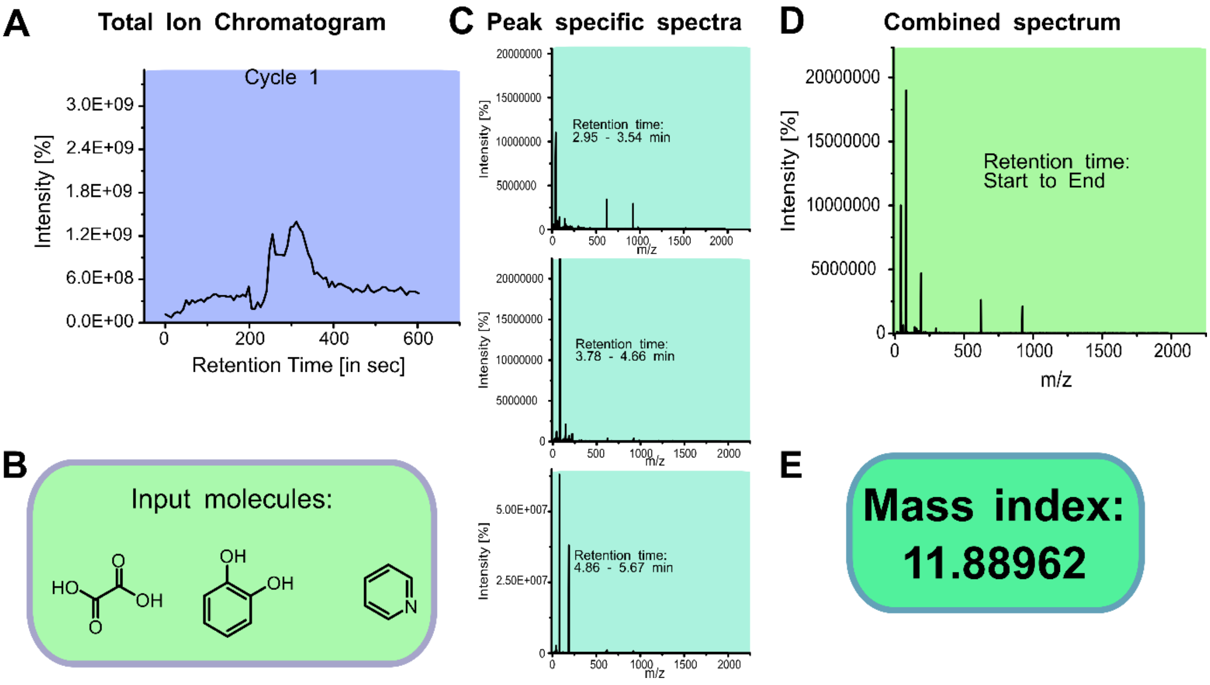

Supplementary Figure 5: Mass Index calculation of cycle 1, the Mass Index is calculated from the sum of all spectra. A presents the TIC of that cycle while B shows the input molecules which have been randomly chosen by the algorithm. In C spectra correlating to peak areas in the TIC are presented. D are all spectra from the TIC combined and this results in E the Mass Index of cycle 1.

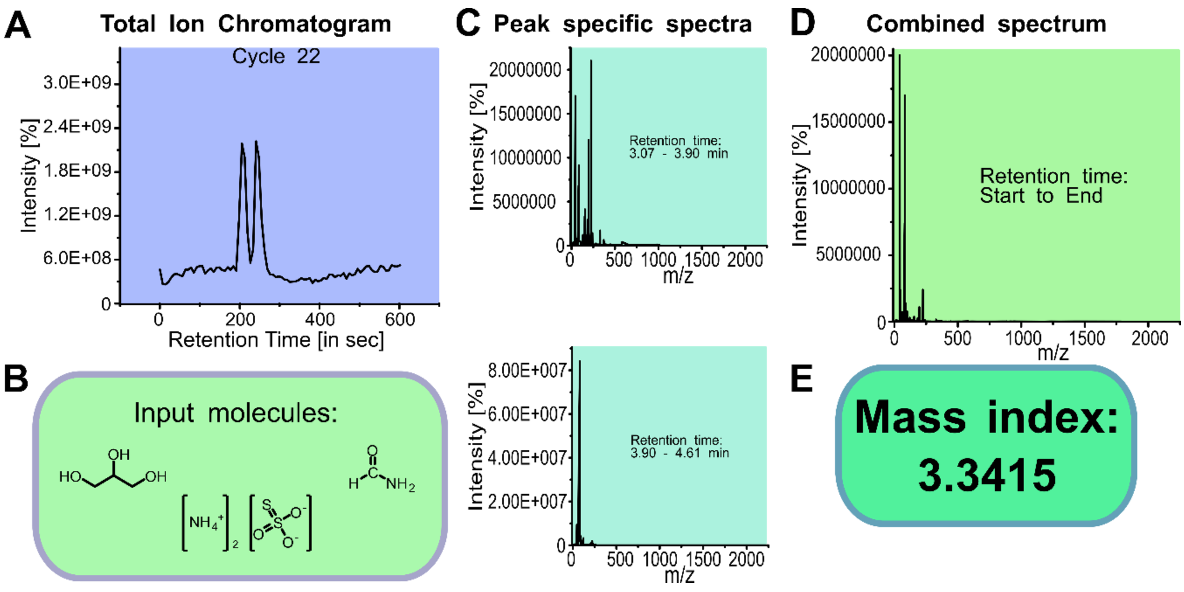

Supplementary Figure 6: Mass Index calculation of cycle 22, the Mass Index is calculated from the sum of all spectra. A presents the TIC of that cycle while B shows the input molecules which have been randomly chosen by the algorithm. In C spectra correlating to peak areas in the TIC are presented. D are all spectra from the TIC combined and this results in E the Mass Index of cycle 22.

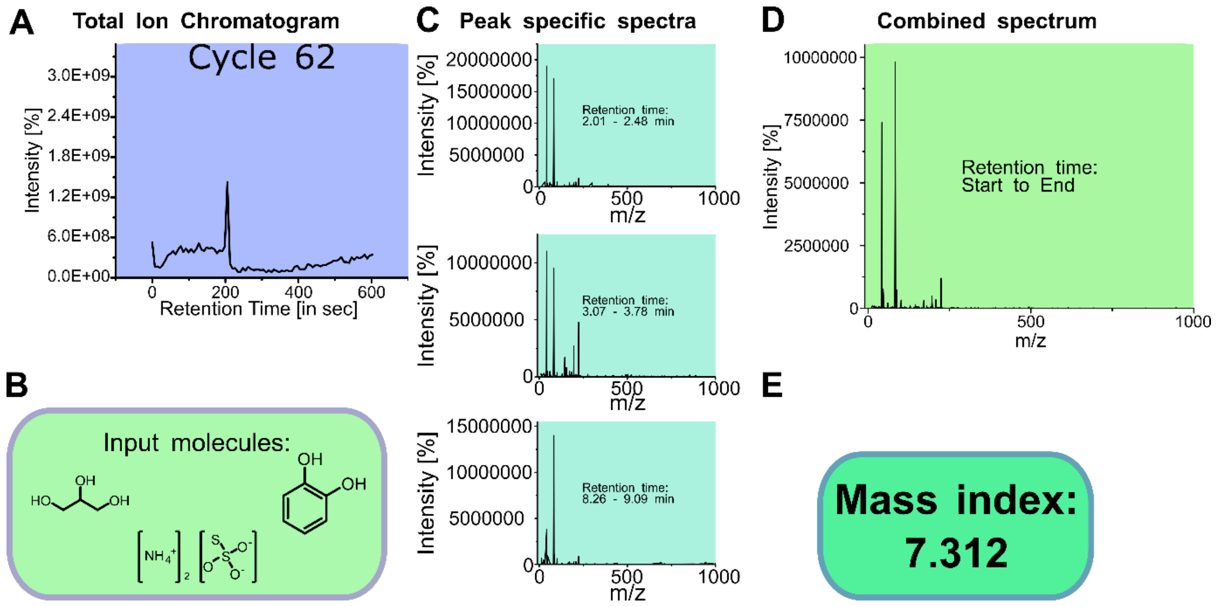

Supplementary Figure 7: Mass Index calculation of cycle 62, the Mass Index is calculated from the sum of all spectra. A presents the TIC of that cycle while B shows the input molecules which have been randomly chosen by the algorithm. In C spectra correlating to peak areas in the TIC are presented. D are all spectra from the TIC combined and this results in E the Mass Index of cycle 62.

4 Example experimental data

4.1 General

All data was collected as described above. As the size of the datasets (over 1000 samples have been collected) do not allow to present every single sample, we show one experiment in detail, as an example for all experiments discussed in the manuscript. This data analysis was complementary, as the main focus of the project was to algorithmically analyse and systematically investigate the experiment instead of screening for every single species which could be found in the analysis. The experiment which was chosen as the example here is referred as Run G in the manuscript.

4.1.1 Input compositions used

Supplementary Table 4: Input solutions, used in Run G. All inputs have been added in the order 1 to 3.

Cycle number	Input 1	Input 2	Input 3
**1-10**	Oxalic acid	Pyridine	Catechol
**11 - 21**	Catechol	Nitric acid	Pyruvic acid

**22-34**	Glycerol	Ammonium thiosulfate	Formamide
**35-44**	Acrylic acid	Potassium pyrophosphate	Glycidol
**45 - 56**	Copper sulfate	Sulfuric acid	Ammonium thiosulfate
**57- 67**	Glycerol	Ammonium thiosulfate	Catechol
**68-75**	Glycidol	Pyridine	Pyruvic acid

4.1.2 pH measurements

Supplementary Table 5: pH values of run G per cycle. Measurement was carried out after the experiment was finished.

Cycle number	pH	Cycle number	pH	Cycle number	pH
**1**	7.90	**24**	6.26	**47**	3.55
**2**	4.80	**25**	6.26	**48**	3.35
**3**	3.87	**26**	6.19	**49**	3.35
**4**	3.33	**27**	6.18	**50**	3.30
**5**	3.05	**28**	6.10	**51**	3.52
**6**	5.71	**29**	6.11	**52**	3.71
**7**	4.78	**30**	6.02	**53**	3.83
**8**	2.45	**31**	6.04	**54**	3.85
**9**	2.67	**32**	6.01	**55**	4.05
**10**	2.82	**33**	6.01	**56**	4.32
**11**	2.44	**34**	6.01	**57**	4.48
**12**	2.08	**35**	5.95	**58**	4.50
**13**	2.89	**36**	5.93	**59**	4.79
**14**	1.83	**37**	5.91	**60**	4.91
**15**	1.81	**38**	5.91	**61**	5.01
**16**	1.83	**39**	5.86	**62**	4.98
**17**	1.84	**40**	5.84	**63**	5.00
**18**	1.81	**41**	5.09	**64**	5.05
**19**	3.90	**42**	5.11	**65**	5.05
**20**	6.49	**43**	4.88	**66**	5.04
**21**	4.52	**44**	4.70	**67**	5.07
**22**	6.47	**45**	3.69	**68**	5.06
**23**	6.37	**46**	3.64	**69**	5.07

4.1.3 Mineral controls

To control the influence of minerals of the experiment, cycles of run G have been manually repeated with and without minerals added.

The experiment has been carried out in 24-hour cycles but based on run G. The first test was the first input set of run G (as described in 4.1) recursed every 24 hours with and without minerals. For the second test, the composition of the replenishing input material was changed in every cycle, e.g. the first input set which was oxalic acid, pyridine and catechol in the first cycle and the second input set catechol, nitric acid and pyruvic acid followed by the third again based on the next input set of run G. This was as well done with and without minerals. The third test have been minerals stirred in water, in which the water was samples and replenished every 24 hours. All experiments have been carried out in triplicates and been measured via ESI-MS as described in 2.5.1. The minerals have been a mix of 1g of ulexite, pyrite and quartz each.

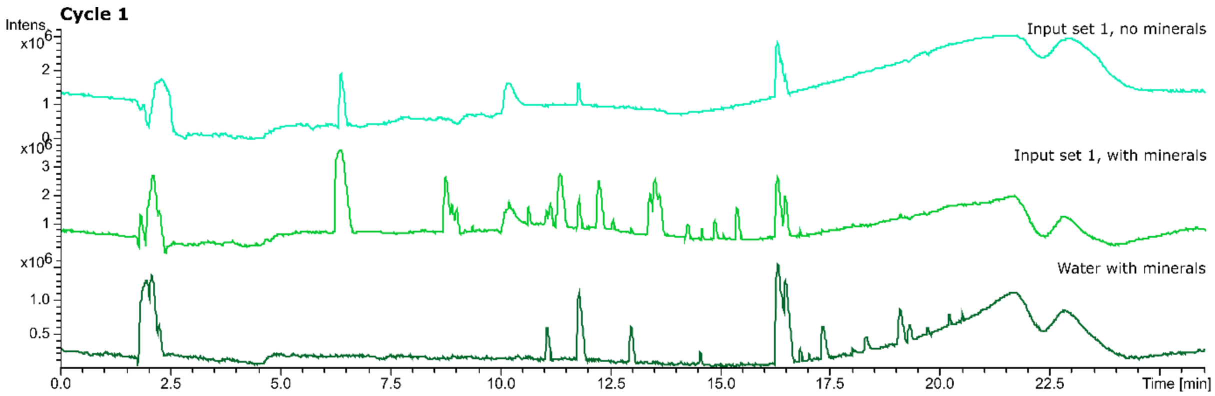

Supplementary Figure 8: Base Peak Chromatograms **(**BPC's) of Cycle 1 of the mineral control run. The graphs top to bottom are 1. The first input composition without minerals, 2. The same input composition with minerals and 3. Water with minerals.

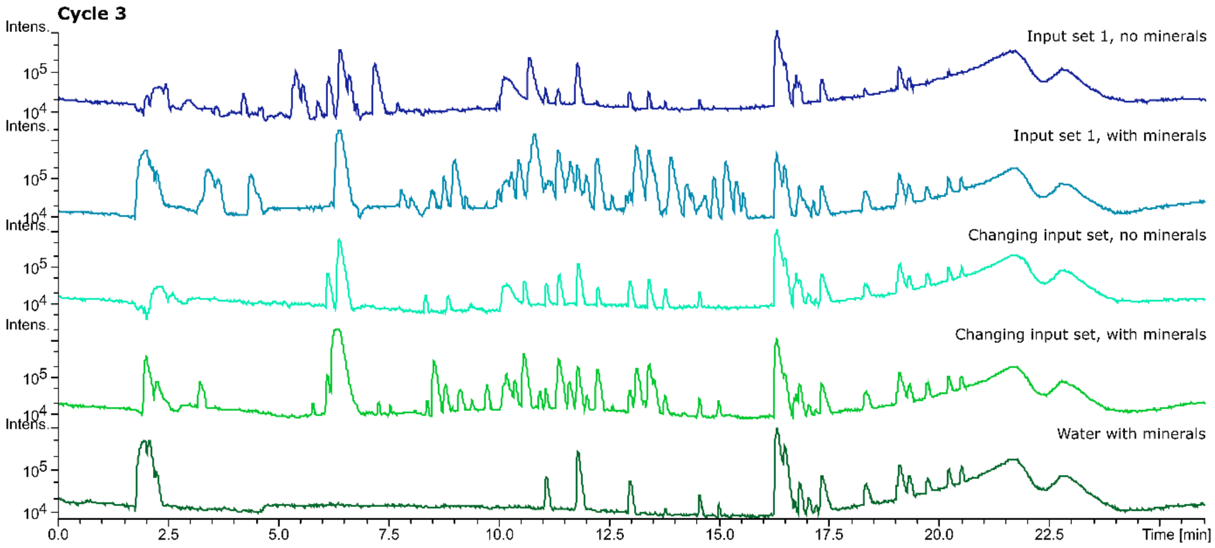

Supplementary Figure 9: BPC's of Cycle 3 of the mineral control run. The graphs top to bottom are 1. The first input composition replenished with the same input set without minerals, 2. The same input composition with minerals and 3. The first 3 input compositions added cycle per cycle, without minerals. 4. The same chemical combination as three, with minerals. 5. Water with minerals.

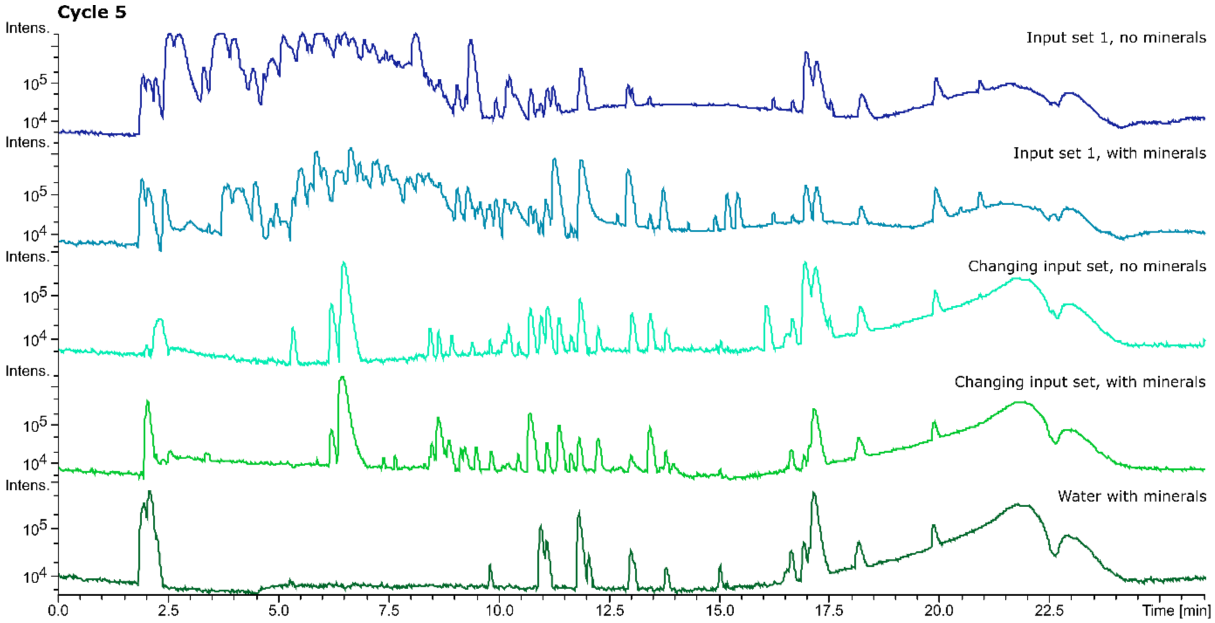

Supplementary Figure 10: BPC's of Cycle 5 of the mineral control run. The graphs top to bottom are 1. The first input composition replenished with the same input set without minerals, 2. The same input composition with minerals and 3. The first 5 input compositions added cycle per cycle, without minerals. 4. The same chemical combination as three, with minerals. 5. Water with minerals.

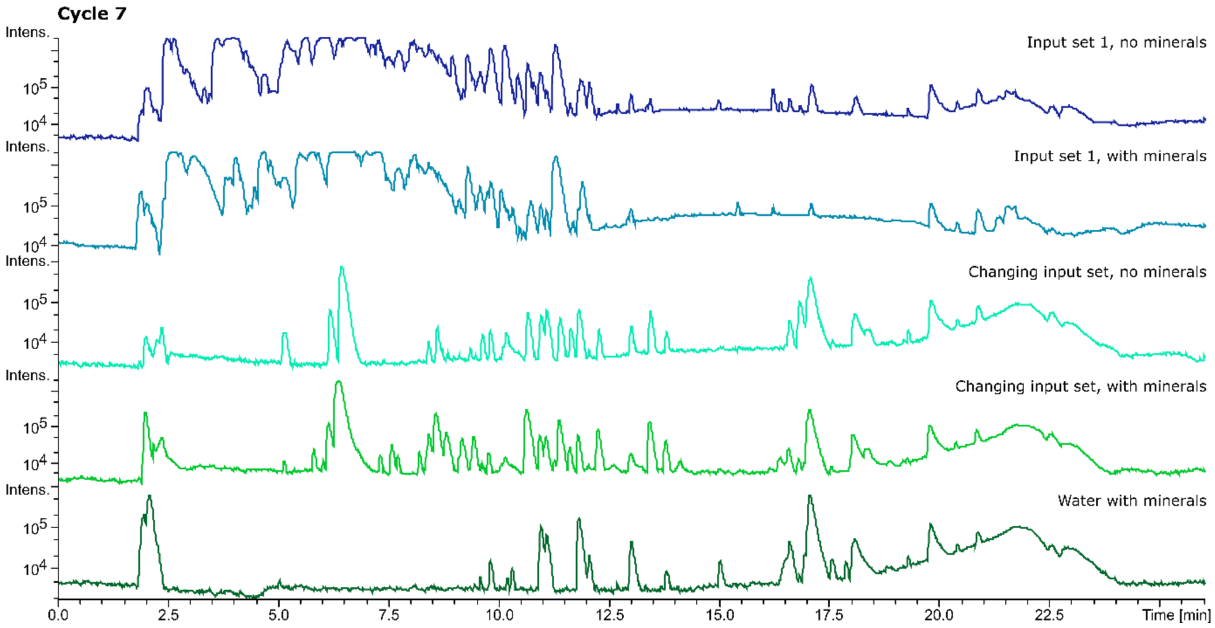

Supplementary Figure 11: BPC's of Cycle 7 of the mineral control run. The graphs top to bottom are 1. The first input composition replenished with the same input set without minerals, 2. The same input composition with minerals and 3. All input compositions of run G added cycle per cycle, without minerals. 4. The same chemical combination as three, with minerals. 5. Water with minerals.

4.1.4 HPLC-DAD data

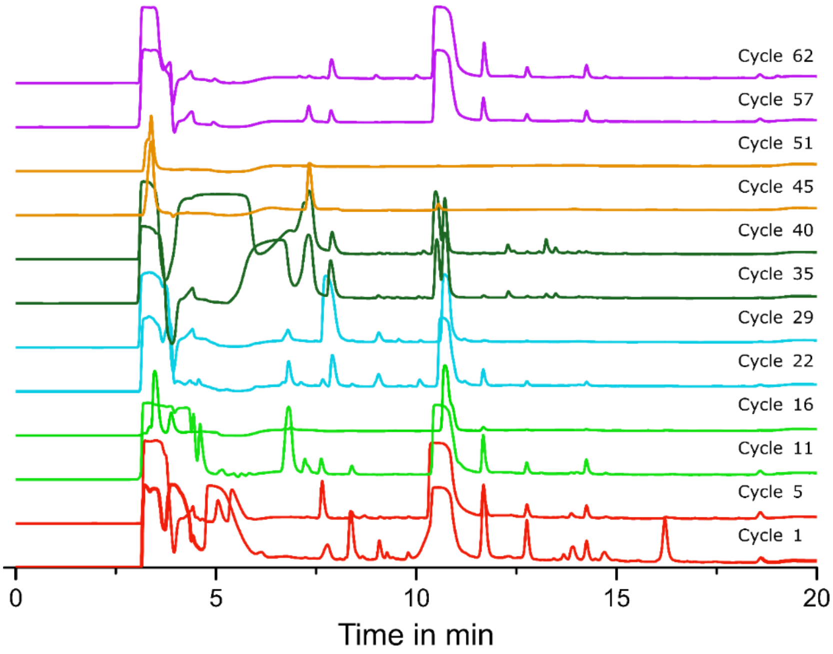

Supplementary Figure 12: HPLC-DAD run overview of Run G. Presented wavelength is 215 nm. The line colour is showing how the different cycles are in context to each other. The cycles with the same line colour, had the identical input sets. The different input sets were: Cycle 1 and 5 (oxalic acid, pyridine, catechol) (red), cycle 11 and 16 (catechol, nitric acid, pyruvic acid) (light green), cycle 22 and

29, (glycerol, ammonium thiosulfate, formamide) (blue), cycle 35 to 40 (glycidol, carbonyldimidazole, oxalic acid) (dark green), cycle 45 and 51 (ethyl acetate, pyruvic acid, ethyl acetate) (orange), cycle 57 and 62 (copper-(II)-sulfate pentahydrate, acrylic acid, glycidol) (purple)

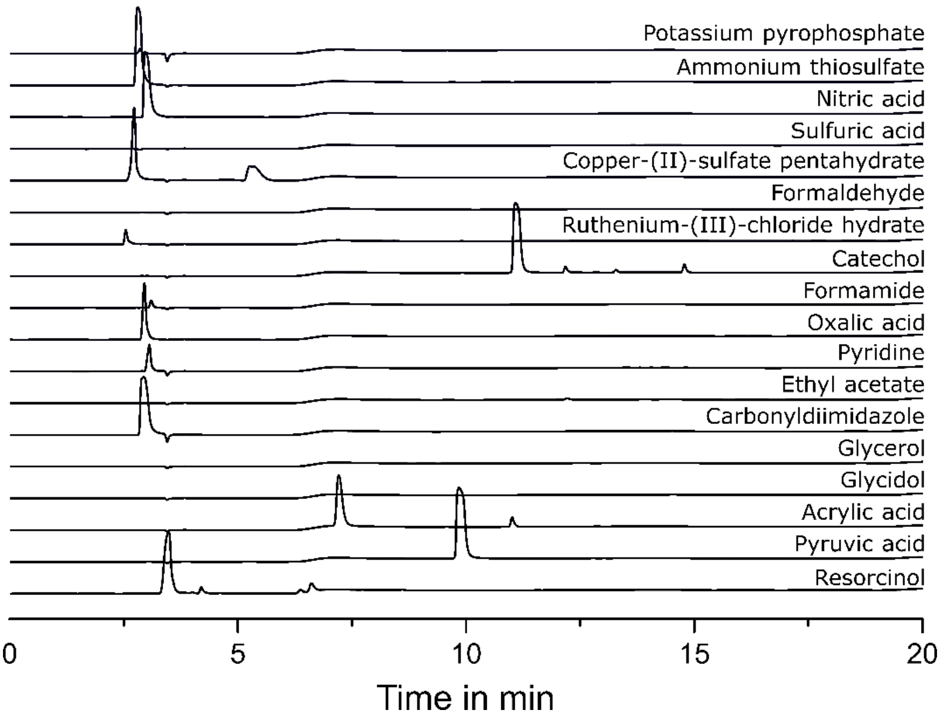

Supplementary Figure 13: Input solutions run in isolation on the HPLC-DAD. All solutions have been exactly prepared as if they have been used as inputs on the platform.

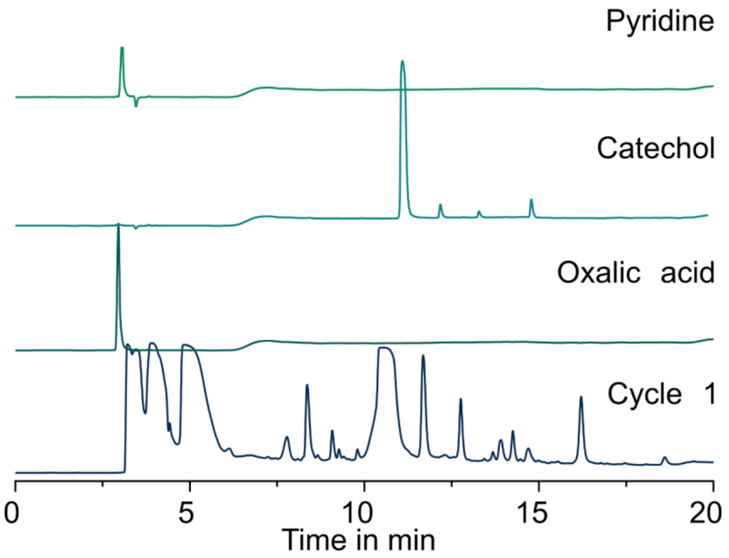

Supplementary Figure 14: Run G cycle 1 compared to the input solutions used in this cycle (oxalic acid, catechol, and pyridine). All standards have been run individual through the same HPLC-DAD.

4.1.5 Online HPLC-MS

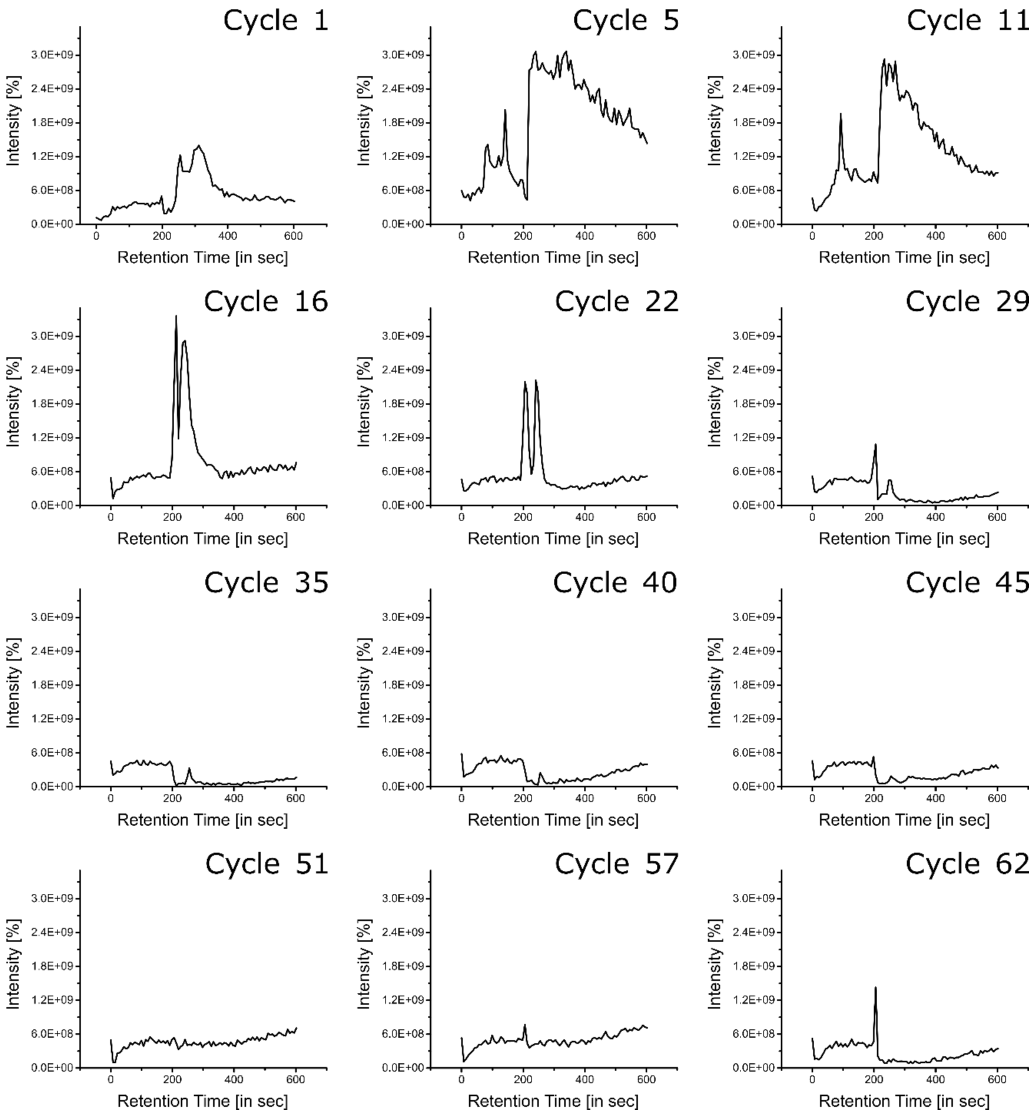

Supplementary Figure 15: Total ion chromatograms of run G from benchtop Advion. The plot shows the intensity of the total signal over the retention time in seconds.

4.1.6 Offline HPLC-ESI-MS

In the following section, we show run G measured on a more sensitive mass spectrometer, a Bruker MAXIS. As we emphasised before, it is not the goal of these experiments to identify every single species but following we show an example approach which could be used for this. However, as this would be too time consuming in reality, does not present a real option.

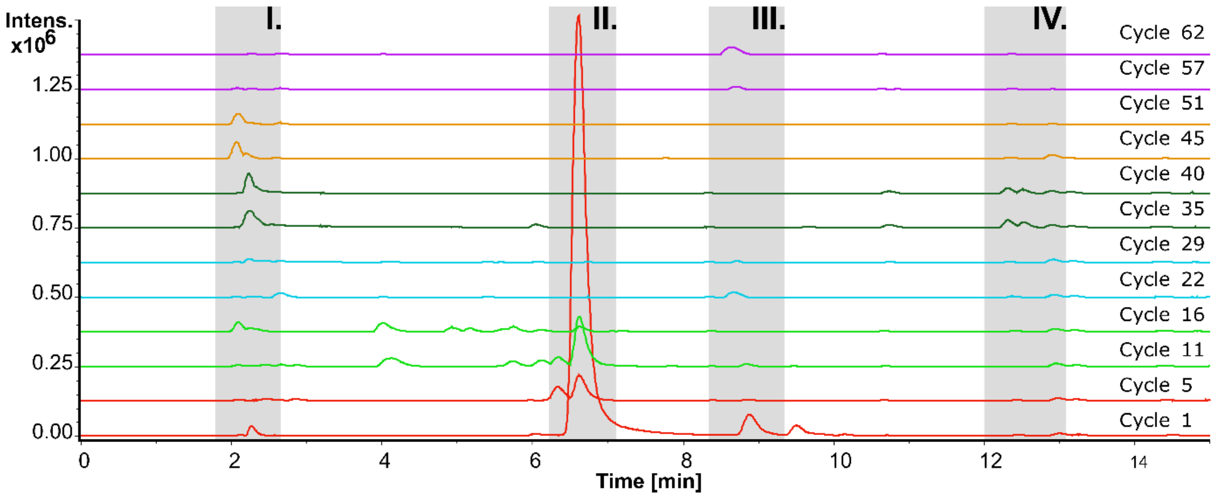

Supplementary Figure 16: BPC chromatograms of offline samples measured on Bruker mass spec compared to each other. The grey areas will be further discussed below.

Many small peaks can be observed in the Bruker BPC. Four retention times are highlighted in grey and are further investigated.

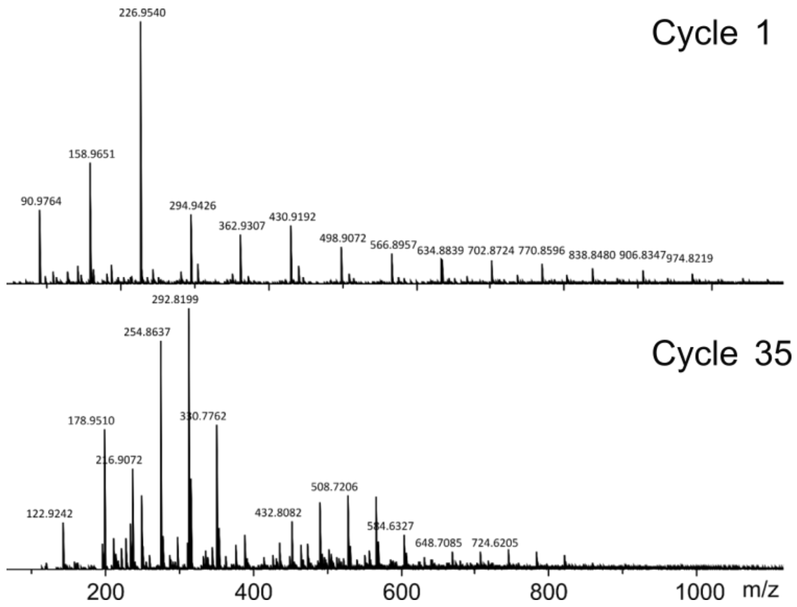

Supplementary Figure 17: Spectra of Bruker chromatogram, cycle 1 and cycle 35 compared in the range from 2.2 to 2.5 minutes, section I of Figure 11. The first peak at 90.97 m/z refers to the oxalic acid of that input set, the higher numbered species might be polymers or products that clustered together.

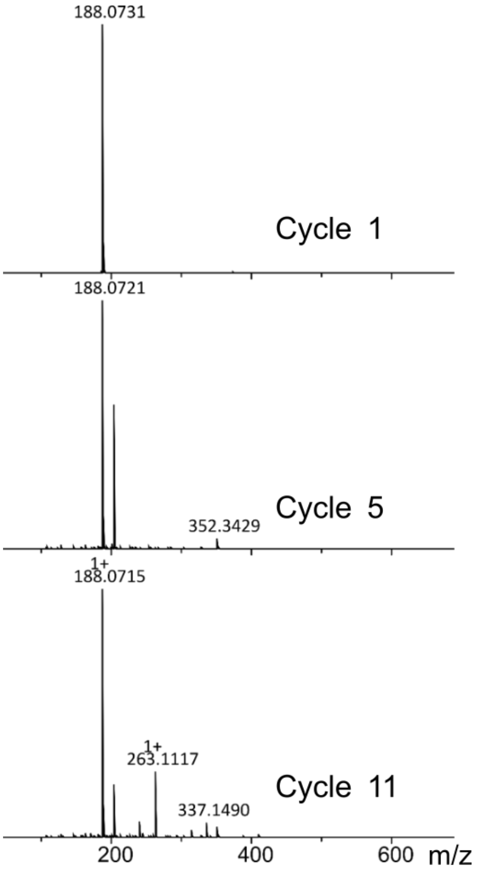

Supplementary Figure 18: Spectra of chromatogram range from 6.5 to 7.2 minutes, section II of Figure 11.

The observed mass of 188.0731 m/z would be consistent with a species with the formula C11H10NO2 and could lead to the hypothesis, that a pyridine molecule reacted with a catechol molecule in a condensation reaction. This reaction appears structurally and energetically very unlikely but the mass spectrometry data confirms the calculated formula. We observe a base peak with the mass of 188.0731 m/z and based on the suggested formula the theoretical mass would be 188.0716 m/z. This means the error is 10.315 ppm. Further to this, when zoomed into the spectra, another peak of an abundance of 12.5 % can be observed (Supplementary Figure 19**:** Spectra of chromatogram range from 6.5 to 7.2 minutes of cycle 1, zoomed in the m/z area between 187 m/z to 190 m/z). This peak at 189.0759 m/z supports the suggested formula, matching the second theoretical most abundant value, with a theoretical abundance of 11.9 % and a value of 189.07452 m/z, resulting in an error of 7.299 ppm. Both errors are low, suggesting that the data is good for the instrument used. As in cycle 1 there is just one peak visible in the shown mass range, while in cycle 5 there are 2 other peaks appearing, one very close to the first one at 204.06 m/z and one peak at 352.34 m/z. Cycle 11 shows more peaks but the 188.07 m/z peak is still standing out and the small peak at 204.06 m/z from cycle 5 is present too. Two new peaks are shown at 263.11 m/z and 337.15 m/z. Neither of the mentioned peaks are matching the mass of any of the starting materials what leads to the conclusion that these peaks relate to products.

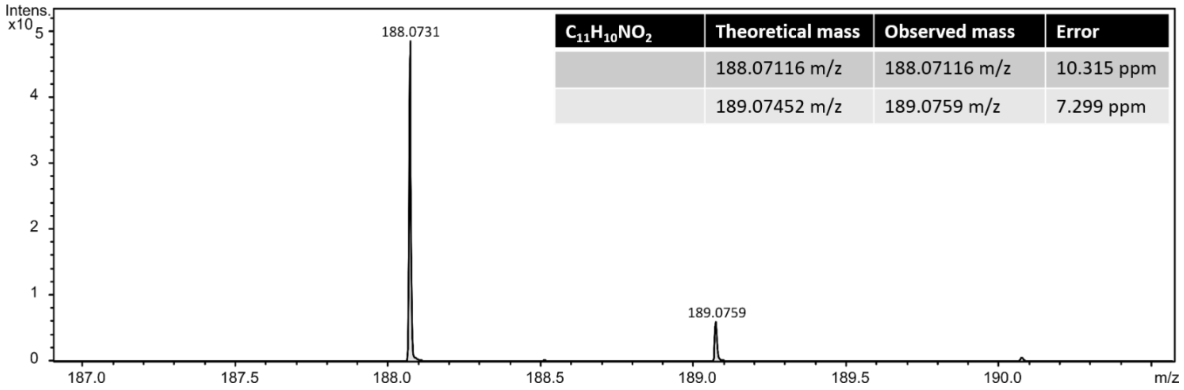

$C_{11}H_{10}NO_2$	Theoretical mass	Observed mass	Error
	188.07116 m/z	188.07116 m/z	10.315 ppm
	189.07452 m/z	189.0759 m/z	7.299 ppm

Supplementary Figure 19: Spectra of chromatogram range from 6.5 to 7.2 minutes of cycle 1, zoomed in the m/z area between 187 m/z to 190 m/z

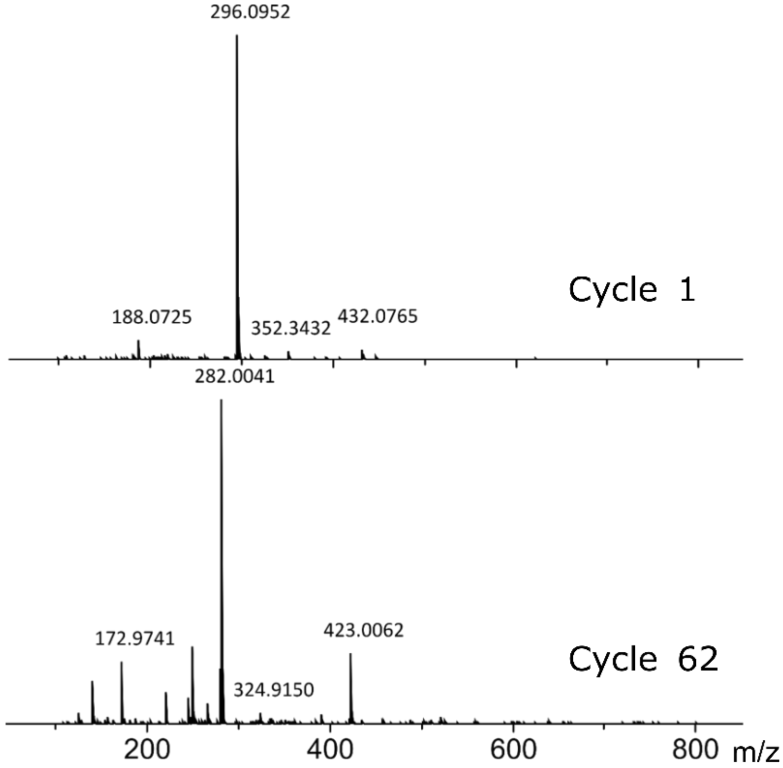

Supplementary Figure 20: Spectra of Bruker chromatogram, range from 8.5 to 8.9 minutes, section III of Figure 11.

Neither of the following spectra have peaks, which we completely identified, the peaks in Supplementary Figure 20 could be related to the catechol input solution or to product species of a catechol related reaction, as these peaks are solely observed if the compound is present in the system. In Supplementary Figure 21**:** Spectra of Bruker chromatogram range from 12.2 to 12.8 minutes, none of the peaks in the spectra relate to input solutions leading to the assumption that these are product species or contaminants.

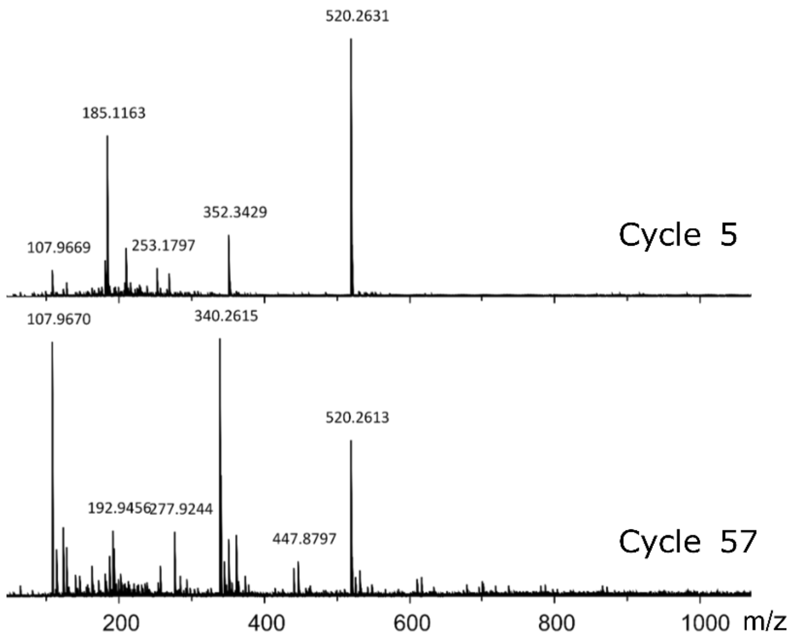

Supplementary Figure 21: Spectra of Bruker chromatogram range from 12.2 to 12.8 minutes, section IV of Figure 11.

4.2 Formula identification

4.2.1 Experimentally found values

Following is a list of found m/z values in the Bruker spectra and proposed molecular formulas based on this value. Differences between the measured m/z value and the monoisotopic mass can be caused by common adducts in positive mode MS like hydrogen, sodium or potassium.

Supplementary Table 6: Measured m/z and proposed correlating formula and monoisotopic mass of selected cycles of run G

Cycle 1			Cycle 5		
**m/z**	**Formula**	**Monoisotopic mass**	**m/z**	**Formula**	**Monoisotopic mass**
107.9671	C3HO3	84.9926	107.9668	C3HO3	84.9926
128.9516	CNO5	105.9776	128.9512	CNO5	105.9776
145.9308	CHNO6	122.9804	145.9304	CHNO6	122.9804
146.9624	C5O4	123.9797	146.9622	C5O4	123.9797
158.9643	CN2O6	135.9756	158.9625	CN2O6	135.9756
163.9417	C5HO5	163.9716	163.9414	C5HO5	163.9716
181.9527	C11HO3	181.9998	181.9523	C11HO3	181.9998
185.1168	C11H16N	162.1283	185.1166	C11H16N	162.1283
186.9583	C7O5	163.9746	186.9579	C7O5	163.9746
188.0733	C11H10NO2	188.2	188.0723	C11H10NO2	188.2
189.0761	C9H12NO2	166.0868	189.0756	C9H12NO2	166.0868
190.0785	C9H13NO2	167.0946	191.106	C12H14O2	190.0994

191.1062	C12H14O2	190.0994	195.101	C12H14N	172.1126
195.1013	C12H14N	172.1126	202.9531	C2H2O11	201.9597
204.0677	C11H9NO3	203.0582	204.0673	C11H9NO3	203.0582
209.1172	C12H16O3	208.1099	204.9709	CH2NO11	203.9628
213.9252	C8HNO5	190.9855	209.1169	C12H16O3	208.1099
217.1072	C10H16O5	216.0998	213.9247	C8HNO5	190.9855
226.9551	C9H2NO5	203.9933	217.1068	C10H16O5	216.0998
256.0632	C12H11NO4	233.0688	226.9566	C9H2NO5	203.9933
257.2506	C12H18NO5	256.1185	257.2499	C12H18NO5	256.1185
283.2278	C13H16NO6	282.0978	283.2276	C13H16NO6	282.0978
296.0955	C12H19NO6	273.1212	285.2816	C17H20N2O2	284.1525
297.0987	C12H20NO6	274.1291	329.3269	C20H18N5	328.1562
326.0703	C11H15N2O8	303.0828	352.343	C17H19N8O	351.1682
329.3275	C20H18N5	328.1562	353.3458	C18H20N6O2	352.1648
352.3435	C17H19N8O	351.1682	354.3451	C16H19N9O	353.1713
353.3464	C18H20N6O2	352.1648	520.2636	C20H19NO14	497.0806
354.3457	C16H19N9O	353.1713			
520.2646	C20H19NO14	497.0806			

**Cycle 10**			**Cycle 15**		
**m/z**	**Formula**	**Monoisotopic mass**	**m/z**	**Formula**	**Monoisotopic mass**
80.0486	C_5H_5N	79.0421	97.9682	CHNO3	74.9956
107.9665	C3HO3	84.9926	107.9664	C3HO3	84.9926
128.9508	CNO5	105.9776	128.9507	CNO5	105.9776
141.9588	C2HNO5	118.9855	141.9587	C2HNO5	118.9855
145.93	CHNO6	122.9804	145.9299	CHNO6	122.9804
146.9616	C5O4	123.9797	146.9616	C5O4	123.9797
158.9619	CN2O6	135.9756	158.0034	C3H5NO5	135.0168
163.9409	C5HO5	163.9716	158.9625	CN2O6	135.9756
181.9517	C11HO3	181.9998	159.9694	C2HO7	136.9722
185.1161	C11H16N	162.1283	163.9407	C5HO5	163.9716
186.9574	C7O5	163.9746	181.0114	C8H4O5	180.0059
188.0716	C11H10NO2	188.2	181.0847	C10H12O3	180.0786
191.1056	C12H14O2	190.0994	181.9515	C11HO3	181.9998
195.1005	C12H14N	172.1126	185.1157	C11H16N	162.1283
202.9523	C2N2O8	179.9655	186.957	C7O5	163.9746
204.0668	C11H9NO3	203.0582	188.0715	C11H10NO2	188.2000
204.9704	C2H2N2O8	181.9811	189.0742	C9H12NO2	166.0868
209.1164	C12H16O3	208.1099	191.1052	C12H14O2	190.0994
213.9243	C8HNO5	190.9855	195.1002	C12H14N	172.1126
217.1063	C10H16O5	216.0998	199.0221	C8H6O6	198.0164
226.9559	C9H2NO5	203.9933	201.0381	C9H8NO3	178.0504
236.9411	C10NO5	213.9776	204.9696	C7H2O6	181.9851
257.2494	C12H18NO5	256.1185	209.116	C12H16O3	208.1099
283.2269	C13H16NO6	282.0978	211.0953	C11H14O4	210.0892

285.2809	C18H36O2	284.2715	213.9237	C8HNO5	190.9855
329.3261	C20H18N5	328.1562	217.1059	C10H16O5	216.0998
352.3422	C17H19N8O	351.1682	231.9344	C8H3NO6	208.9960
353.345	C18H20N6O2	352.1648	236.9404	C6H2N2O7	213.9862
354.3445	C16H19N9O	353.1713	246.8639	C10NO7	245.9675
520.2627	C20H19NO14	497.0806	254.951	C10H2NO6	231.9882
			257.249	C12H18NO5	256.1185
			263.112	C11H18O7	262.1053
			264.8744	C11NO6	241.9726
			282.8852	C10N2O7	259.9706
			283.226	C13H16NO6	282.0978
			285.2803	C18H36O2	284.2715
			305.9014	C13HNO7	282.9753
			329.3253	C20H18N5	328.1562
			352.3413	C17H19N8O	351.1682
			353.3441	C18H20N6O2	352.1648
			354.3433	C16H19N9O	353.1713
			520.2611	C20H19NO14	497.0806
			520.7628	C20H2N8O9	497.9945
**Cycle 20**			**Cycle 25**		
**m/z**	**Formula**	**Monoisotopic mass**	**m/z**	**Formula**	**Monoisotopic mass**
107.9664	C3HO3	84.9926	107.9663	CHNO3S	106.9677
115.0364	C3H8O3	92.0473	115.0363	C3H8O3	92.0473
128.9506	CNO5	105.9776	128.9506	CNO3S	105.9599
141.9587	C2HNO5	118.9855	145.9299	CHNO2S2	122.9449
145.9299	CHNO6	122.9804	146.9615	CH2NO4S	123.9705
146.9616	C5O4	123.9797	163.9406	CHO6S	140.9494
158.0032	C3H5NO5	135.0168	171.0633	C6H12O4	148.0736
163.9406	C5HO5	163.9716	173.079	C8H12O4	172.0736
171.0634	C9H10NO	148.0762	181.0846	C6H16N2S2	180.0755
181.9515	C11HO3	181.9998	181.9515	C3HO7S	180.9443
185.1157	C11H16N	162.1283	185.1157	C10H16O3	184.1099
186.957	C7O5	163.9746	186.9571	C5H2N2O2S2	185.9558
191.1052	C12H14O2	190.0994	191.105	C7H14N2O4	190.0954
192.9802	C2H4NO8	169.9937	195.0999	C7H18N2S2	194.0911
195.1001	C12H14N	172.1126	204.9681	C5H4N2O3S2	203.9663
204.968	C7H2O6	181.9851	209.1159	C10H18O3	186.1256
209.116	C12H16O3	208.1099	213.9237	C3HO7S2	212.9164
213.9236	C8HNO5	190.9855	217.1059	C10H16O5	216.0998
217.1059	C10H16O5	216.0998	231.9346	C4H3NO5S2	208.9453
236.9405	C6H2N2O7	213.9862	236.9404	C6H2N2O3S2	213.9507
238.0149	C16HN2O	237.0089	254.9509	C6H4N2O4S2	231.9612
239.022	C13H4NO4	238.014	257.2489	C8H20N2O7	256.1271
254.9513	C10H2NO6	231.9882	282.0032	C8H11NO6S2	281.0028

257.2488	C12H18NO5	256.1185	283.2259	C9H18N2O6S	282.0886
282.0033	C14H3NO6	280.996	329.3254	C20H18N5	328.1562
283.0282	C6H8N3O10	282.021	352.3413	C17H19N8O	351.1682
283.2261	C13H16NO6	282.0978	353.344	C18H20N6O2	352.1648
329.3254	C20H18N5	328.1562	520.2613	C20H19NO14	497.0806
352.3413	C17H19N8O	351.1682	520.7629	C20H2N8O9	497.9945
353.3443	C18H20N6O2	352.1648			
354.3432	C16H19N9O	353.1713			
520.2613	C20H19NO14	497.0806			

**Cycle 30**			**Cycle 35**		
**m/z**	**Formula**	**Monoisotopic mass**	**m/z**	**Formula**	**Monoisotopic mass**
107.9662	CHNO3S	106.9677	107.9662	CO4P	106.9534
115.0363	C3H8O3	92.0473	115.0362	C5H6O3	114.0317
128.9505	CNO3S	105.9599	122.9243	CO3P2	121.9323
145.9299	CHNO2S2	122.9449	128.9505	H2O4P2	127.9428
158.0032	C6H5O3S	156.9959	145.9299	C2H4KOP2	144.9374
163.9407	CHO6S	140.9494	163.9407	C3HO4P2	162.9350
171.0634	C6H12O4	148.0736	167.0319	C8H6O4	166.0266
173.079	C8H12O4	172.0736	178.9512	C4H5KO3	139.9876
178.9514	C4H2O4S2	177.9395	181.0847	C10H12O3	180.0786
181.0848	C6H16N2S2	180.0755	181.9514	C3H3O5P2	180.9456
181.9515	C3HO7S	180.9443	185.1157	C10H16O3	184.1099
185.1157	C10H16O3	184.1099	186.957	C2H4O6P2	185.9483
186.9572	C5H2N2O2S2	185.9558	191.1051	C12H14O2	190.0994
190.8646	CH2O7S2	189.9242	195.1	C9H16O3	172.1099
191.1051	C7H14N2O4	190.0954	200.9329	C2H2O7P2	199.9276
192.9804	C5H4O6S	191.9729	201.9276	C2H3K2O6	200.9204
195.1001	C7H18N2S2	194.0911	204.9677	C4H6KO5P	203.9590
201.9277	C3HNO4S2	178.9347	209.1158	C12H16O3	208.1099
204.9679	C5H4N2O3S2	203.9663	210.9333	C6H5K3O	209.9252
209.1159	C10H18O3	186.1256	212.8519	C2HK4P	211.8364
210.9335	C2H4O6S2	187.9449	213.9238	CH3KO8P	212.9203
213.9239	C3HO7S2	212.9164	216.9074	C2H3K2O5P	215.8992
216.9078	C2H2NO7S2	215.9273	217.1059	C10H16O5	216.0998
217.1059	C10H16O5	216.0998	224.0929	C12H16O2P	223.0888
228.821	C5H2O5S2	205.9344	228.8207	HK3O3P2	227.8312
236.9405	C6H2N2O3S2	213.9507	236.9404	C6H7K2O3P	235.9407
239.0537	C7H14N2O3S2	238.0446	239.0539	C11H10O6	238.0477
254.8642	C8NO5S2	253.9218	254.8639	C2H2K3O5P	253.8551
254.9511	C6H4N2O4S2	231.9612	257.2489	C9H20O8	256.1158
257.249	C8H20N2O7	256.1271	266.9269	C5H11K3OP2	265.9196
263.112	C10H18N2O4S	262.0987	277.8692	C6HK2O4P2	276.8624
277.8694	C6HN2O7S2	276.9225	283.2262	C10H18O9	282.0951

283.2262	C9H18N2O6S	282.0886	292.8202	C2H2K4O3P2	291.8027
295.88	C10HNO6S2	294.9245	294.8189	C5HK3O2P2	271.8363
318.8965	C10H8NO7S2	317.9742	295.8797	C6H3K2O5P2	294.8730
323.9116	C10H13NO7S2	323.0133	318.8961	C9H6K4O3	317.8865
342.7669	C10N4O5S2	319.931	330.7765	C2H2K4O4P2	307.7977
352.3415	C17H19N8O	351.1682	345.0635	C12H17KO9	344.0510
353.3444	C18H20N6O2	352.1648	352.3415	C20H40KO2	351.2665
415.9604	C9H11N4O7S4	414.9511	353.3445	C17H36O7	352.2461
422.7932	C10H12N2O7S4	399.9527	422.7929	C13H2K2P6	421.7856
520.2614	C20H19NO14	497.0806	432.8086	C4H11KO4P8	409.8195
520.7633	C20H2N8O9	497.9945	470.7652	C6H6K4O7P4	469.7612
			508.7212	C13H2K4O2P4	469.7554
			520.2615	C20H39O15	519.2289
			546.6773	C2H2K4O15P4	545.6892
**Cycle 40**			**Cycle 45**		
**m/z**	**Formula**	**Monoisotopic mass**	**m/z**	**Formula**	**Monoisotopic mass**
107.9662	CO4P	106.9534	107.9661	C2H3OS2	106.9625
115.0362	C5H6O3	114.0317	123.9404	C2H4CuS	122.933
128.9504	H2O4P2	127.9428	128.9504	C2H3O2P	89.9871
145.9296	C2H4KOP2	144.9374	141.9585	C3H4O2P	102.9949
163.9405	C3HO4P2	162.9350	145.9296	CH2CuNO2	122.9382
167.0316	C8H6O4	166.0266	159.9692	C2H5NO2P2	136.9796
174.8872	H2K2O2P2	173.8804	163.9404	CH3NO4S	124.9783
178.9511	C4H5KO3	139.9876	174.8868	K2O4S	173.8791
181.0846	C10H12O3	180.0786	181.9512	CH7CuKN2O2	180.9441
181.9514	C3H3O5P2	180.9456	186.9567	C3H8OP2S2	185.9492
185.1155	C10H16O3	184.1099	204.9674	C5H10KPS2	203.9599
186.9569	C2H4O6P2	185.9483	213.9234	CH7CuKN2O2S	212.9161
191.1049	C12H14O2	190.0994	228.1966	C6H15N2O7	227.0879
195.0998	C9H16O3	172.1099	246.8635	C2H4K4NOS	245.8562
204.9676	C4H6KO5P	203.9590	257.2485	C12H35NO2P	256.2405
209.1156	C12H16O3	208.1099	264.8742	C6H4KPS3	241.885
210.933	C6H5K3O	209.9252	282.885	C5H10K2S3	243.9219
212.8435	HK3O2P2	211.8363	283.2255	C9H34N2O5S	282.2188
213.9236	CH3KO8P	212.9203	287.8902	C3H9NO2P2S3	248.9271
214.8412	CHKOP4	191.8615	305.9008	C8H7KNPS3	282.9115
216.9072	C2H3K2O5P	215.8992	310.8114	C3H3K4NOPS	287.8221
217.1057	C10H16O5	216.0998	352.3408	C12H35N2O9	351.2343
239.0537	C11H10O6	238.0477	353.3436	C8H36N2O12	352.2268

254.8636	C2H2K3O5P	253.8551	520.2601	C10H35N2O21	519.1732
257.2486	C9H20O8	256.1158	520.7616	CH21CuK4N2OS7	497.7724
277.8691	C6HK2O4P2	276.8624			
283.2258	C10H18O9	282.0951			
292.8198	C2H2K4O3P2	291.8027			
295.8796	C6H3K2O5P2	294.8730			
310.8118	C2H5K4O2P3	309.8051			
345.0632	C12H17KO9	344.0510			
352.3413	C20H40KO2	351.2665			
353.3441	C17H36O7	352.2461			
390.7886	C5HKO8P4	351.8259			
428.7445	C6H6K4O2P4	389.7867			
520.2611	C20H39O15	519.2289			
**Cycle 50**			**Cycle 55**		
**m/z**	**Formula**	**Monoisotopic mass**	**m/z**	**Formula**	**Monoisotopic mass**
97.9683	HO4S	96.9596	107.9668	HN3S2	106.9612
107.9664	HN3S2	106.9612	115.0368	H4NO4S	113.9861
113.9634	H4CuNO2	112.9538	123.9412	H6CuO2	100.9664
123.9407	H6CuO2	100.9664	128.9511	H3CuNO3	127.9409
128.9507	H3CuNO3	127.9409	141.9592	H3N3S3	140.9489
141.9588	H3N3S3	140.9489	145.9303	HO5S2	144.9265
145.9298	HO5S2	144.9265	163.9411	H9Cu2N2	162.9358
146.9615	N2O6	123.9756	181.9519	H11Cu2N2O	180.9463
159.9695	H5N3OS3	158.9595	186.9573	H9CuN2O2S	163.9681
163.9406	H9Cu2N2	162.9358	192.9458	H6N3OS4	191.9394
181.9513	H11Cu2N2O	180.9463	204.9693	H4N4O5S2	203.9623
186.9568	H9CuN2O2S	163.9681	228.1974	H19N3O7S	205.0944
192.9452	H6N3OS4	191.9394	257.2491	H28N6O9	256.1918
204.9676	H4N4O5S2	203.9623	277.9238	H11Cu2N2O7	276.9158
213.9237	H4CuN2O6	190.9365	282.8857	H5CuO8S2	259.8722
228.1967	H19N3O7S	205.0944	352.3413	H39N4O16	351.2361
246.8636	H6Cu2N2O2S	223.8742	353.3441	H40N4O16	352.2439
257.2486	H28N6O9	256.1918			
264.8743	H9CuO4S4	263.868			
276.898	H7CuNO8S2	275.8909			
277.9229	H11Cu2N2O7	276.9158			
282.8851	H5CuO8S2	259.8722			
287.8904	H2CuNO7S3	286.8289			
305.901	H4CuNO8S3	304.8395			
352.3408	H39N4O16	351.2361			
353.3438	H40N4O16	352.2439			
**Cycle 60**			**Cycle 65**		

m/z	Formula	Monoisotopic mass	m/z	Formula	Monoisotopic mass
107.9673	C2H3OS2	106.9625	107.9674	C2H3OS2	106.9625
115.0373	C5H6O3	114.0317	115.0374	C5H6O3	114.0317
123.9416	CHNO2S2	122.9449	123.9416	CHNO2S2	122.9449
128.9516	CH2N2OS	89.9888	128.9517	CH2N2OS	89.9888
145.9308	CHNO3S	106.9677	145.9310	CHNO3S	106.9677
146.9626	CH4N2O2S	107.9993	163.9418	CH3NO4S	124.9783
163.9416	CH3NO4S	124.9783	181.9527	C4HNO4S	158.9626
181.9525	C4HNO4S	158.9626	186.9582	C2H4NO5S2	185.9531
186.9579	C2H4NO5S2	185.9531	192.9464	C5H2N2O2S	153.9837
192.9463	C5H2N2O2S	153.9837	199.0235	C8H6O6	198.0164
204.9703	C3H6N2O3S2	181.982	228.1983	C7H17NO7	227.1005
228.1982	C7H17NO7	227.1005	257.2504	C8H20N2O7	256.1271
238.0158	C8H9NO4S	215.0252	277.9248	C8H3N2O2S3	254.9357
250.0318	C9H9NO6	227.043	282.0046	C7H11N3O3S3	280.9963
257.2502	C8H20N2O7	256.1271	352.3430	C15H29N4OS	313.2062
277.9249	C8H3N2O2S3	254.9357	353.3459	C16H36N2O6	352.2573
282.0042	C7H11N3O3S3	280.9963			
352.3427	C15H29N4OS	313.2062			
353.3456	C16H36N2O6	352.2573			

4.2.2 Matching experimental and theoretical formulas

The network model described in the main manuscript, predicted 2206 possible reactions with the used input library. The theoretical number of the resulting products of these reactions is 429 (multiple reactions can lead to the same product). These products have been checked with the proposed experimentally assigned formula above in Supplementary Table 6 and 22 matching structures have been identified as listed below in Supplementary Table 7.

Supplementary Table 7: Matching formulas of experimentally and theoretical found structures

FORMULA	MOLECULAR WEIGHT	COMPOUND NAME
**C3H9NO2**	91.11	3-aminopropane-1,2-diol
**C6H8N+**	94.13	1-Methylpyridinium
**C3HNO4**	115.04	Iminomalonate
**C2H5NO**	59.07	Acetamide
**H2O7P2-2**	175.96	dihydrogen diphosphate
**C3H4O2**	72.06	3-Oxetanone
**C2H5NO2**	75.07	Methyl carbamate
**C5H4O6**	160.08	2,4-Dioxopentanedioic acid
**N2O5**	108.01	Dinitrogen pentoxide
**O4P-3**	94.971	orthophosphate

**C8H6O4**	166.13	6,7-Dihydroxycoumaranone
**C5H4N2O3**	140.1	5-Formyluracil
**CH2N2**	42.04	Cyanamide
**C6H4N2**	104.11	3-Cyanopyridine
**C3H5NO**	71.08	Acrylamide
**C3H5NO5**	163.13	3-(Nitrooxy)propanoic acid
**C5H4N2**	92.1	Pyrrole-2-carbonitrile
**C6H12O4**	148.16	1-O-Methyl-2-deoxy-D-ribose
**C9H12NO2+**	166.2	1-carboxy-2-phenylethanaminium
**C3H8O3**	92.09	1,1,2-Propanetriol
**C11H10NO2+**	188.2	indole-3-propanoate
**C5H6O3**	114.1	4,5-Dimethyl-1,3-dioxol-2-one

4.2.3 Product persistence over dilution

The product peaks shown above in 4.2.2 have been tracked by their intensity over the duration of the run time. Supplementary Figure 22 shows that some product species do persist throughout the run while others are being diluted out after a change in the input composition.

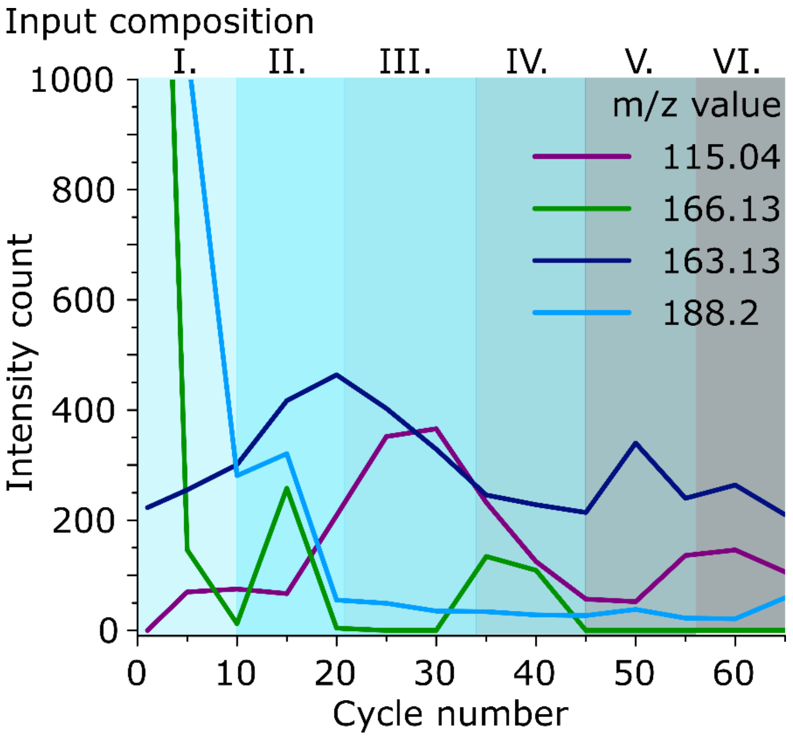

Supplementary Figure 22: Comparison of product peaks of run G. The purple line shows a peak not present in cycle 1 but rising through the whole run up from cycle 2. The green line shows a peak which is high in the initial cycle but is not persisting throughout the run. The dark blue line shows a product peak, present through the full run and the pale blue line shows a peak which is high with the initial input composition but decreasing throughout the run.

5 Decision Making Algorithm

5.1 Architecture

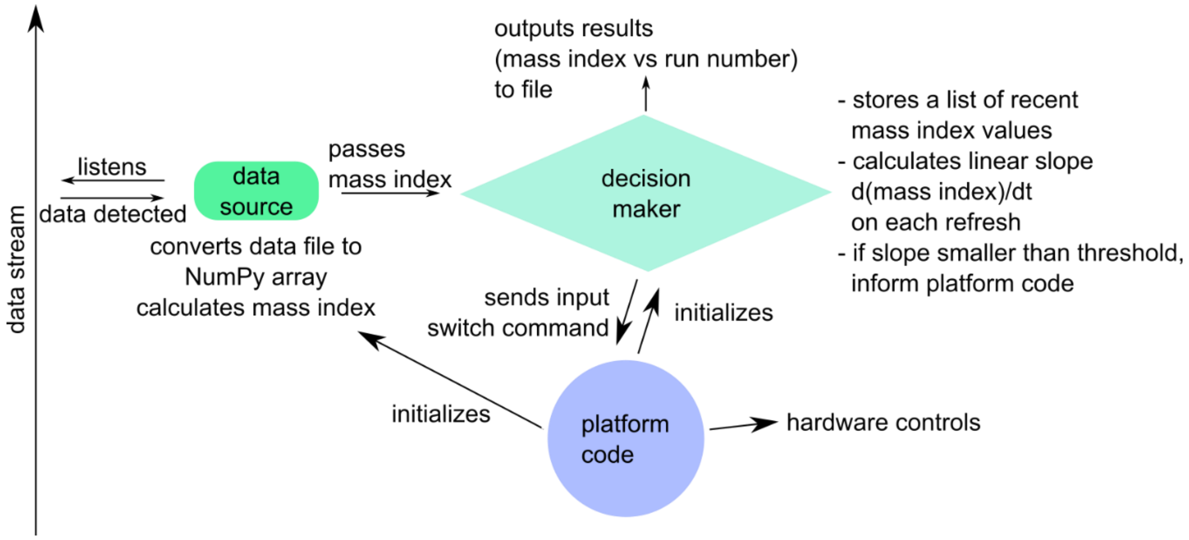

Supplementary Figure 23: The general architecture of the software controls of the recursive reactor responsible for the background work. The platform code (dark blue) controls the overall experiment, initialises the analysis and manages the hardware control. The data source (green) takes the data from the Advion MS and calculates it into a value which the decision maker (pale blue) can interpret and pass back to the platform code for experiment adjustments.

The control software, written in Python 3, is designed to be modular as well as extensible and incorporates data analysis functionality spread over several Python classes (Supplementary Figure ***23***). The data source in the form of the DataSource class is responsible for monitoring the directory in which the analytical data is saved for changes, calculating the appropriate measure of complexity and exposing it to the decision-making algorithm. The DataSource class was designed to utilize various data types and formats; in addition to the LC-MS data used in the present work, other inputs such as chromatography and nuclear magnetic resonance spectroscopy could also be employed.

The decision-making itself is the responsibility of the DecisionMaker class, which implements the algorithm described below; based on the outcome, it sends the appropriate command to the main hardware control loop, as well as saving the results of the calculations to a file for any required offline analysis and plotting.

The main hardware control loop is tasked with interfacing with all the physical components of the system: the stirrer/heater, the pumps and the valves. Whenever a run is started, it also initializes the data source, specifying the directory in which to wait for new data, as well as starting the decision maker in a separate thread to prevent blocking.

5.2 Measure of complexity

As mentioned above, the measure of complexity will be specified differently depending on the data format and the analytical technique used. For the bulk of the present study, we focused on the mass spectrometry data and on the so-called Mass Index, used by our group earlier for monitoring complexity changes in amino acid mixtures.

The Mass Index is a real number carrying information on the number of peaks and the mass range in a given mass spectrum. Since each data point in our case was a total ion current (TIC) chromatogram recorded by the LC-MS equipment, a modified approach was required. Upon successful acquisition, the data was exported on-line to the NetCDF file format. This was read using the Python library `netCDF4` to convert it to an in-memory array accessible to Python. The resulting array could be thought of as a stack of mass spectra, each entry corresponding to a certain retention time. For each mass spectrum, the Mass Index was calculated by discarding all peaks whose intensity was less than 10^6, subtracting the lowest m/z value in the spectrum from the highest, and dividing the result by the number of peaks. If there were no peaks left after applying the intensity thresholding, the value 0.0 was returned. The general visual idea for the process of extracting the Mass Index is presented in Supplementary Figure **24.**

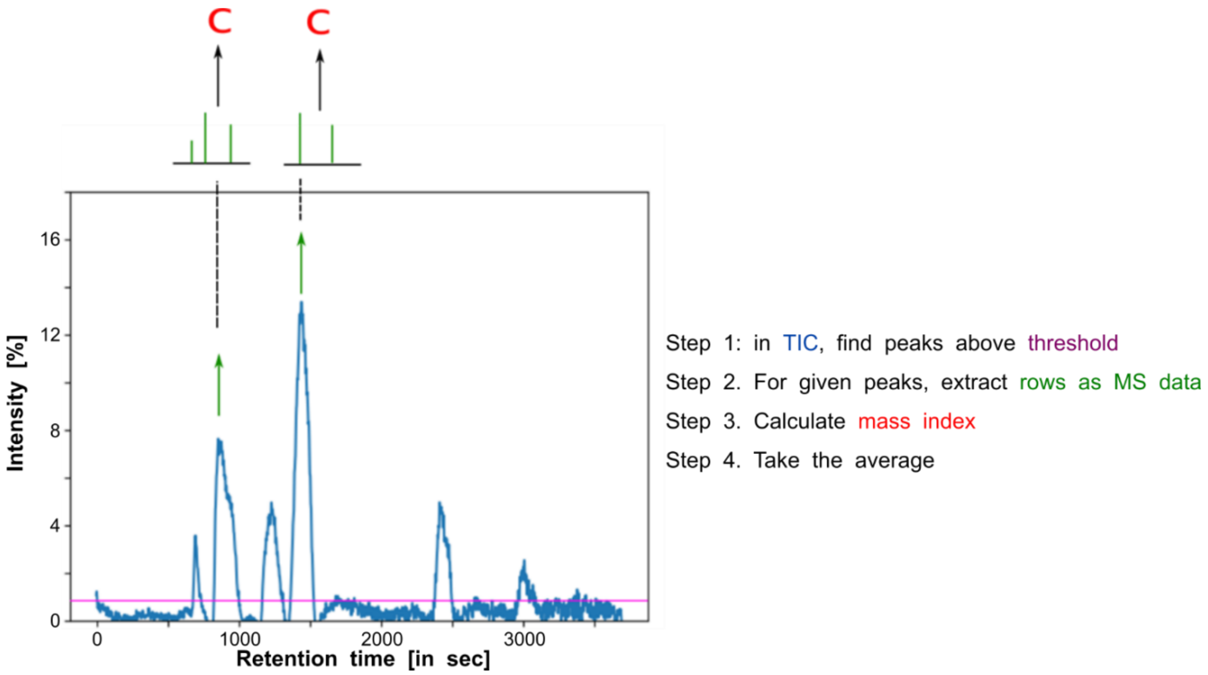

Supplementary Figure 24: Conceptual scheme of the steps required to convert the total ion chromatograms (TICs) recorded on-line during system operation to individual numbers, i.e. mass indices.

The obtained list of mass indices for the individual components of the TIC was then averaged, and the result was passed on to the decision-making algorithm as a complexity measure.

5.3 Decision making algorithm example

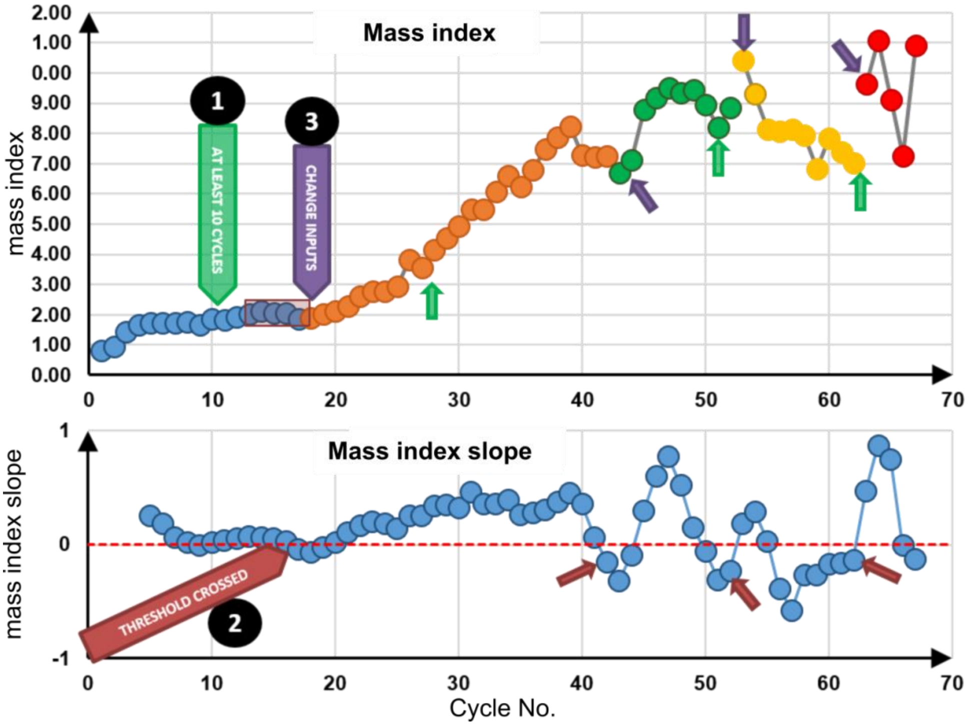

Supplementary Figure 25: Concept of assigning change, visualised with real data of a run assigned with the Mass Index. The top graph shows the Mass Index of each cycle with each input set in a different colour, while the graph in the bottom shows the slope between the Mass Index values of the cycles

The role of the algorithm is to detect when the complexity of the reaction mixture has stopped increasing and, whenever that happens, to send a signal to the hardware control loop telling it to change the chemical inputs to a new random set. It receives the successive Mass Index values and stores them in a Python list. It takes two external parameters, the change threshold and the change interval. The threshold parameter specifies the critical value of the slope in the time dependency of the Mass Index below which the algorithm assumes that the values have plateaued. In the current work, this was set to a small positive value, 0.01. The change interval represents the minimum number of data points deemed to be sufficient for meaningful slope calculation. In our case, the slope was taken over 5 most recent points, as long as at least 10 data points have accumulated since either the start of the run or the last input switch.

Each time a new data point is detected and the corresponding m/z index measured, the algorithm first checks if the condition above is met – if it is not, it waits for the next data point. As soon as the 10th data point arrives, the slope is taken using simple linear regression against the vector [0, 1, 2, 3, 4]. When the slope is above the threshold specified above, the least recent data entry is removed from the list, replaced with the one just recorded and the algorithm waits for the next data point. For the slopes below the threshold, the DecisionMaker class sets the relevant boolean flag in the configuration file to

True. At the start of the subsequent cycle, when the hardware loop detects that flag, it proceeds to the next entry in its list of randomly generated chemical input sets.

5.4 Alternative algorithms tested

Based on the issues that have been highlighted, with the initial used mz algorithm, alternative ways to evaluate the data were found. As one of the problems of the Mass Index, was the fact that there was no relation between the mass of the peak and its intensity, it was interesting to investigate how the data would change if the intensity would be multiplied by the mass of the corresponding peak.

5.4.1 Adaptive Mass Index

An adaptive formula was developed to check whether the original mass index was sensitive to 1) the hard threshold used, and 2) outliers at the higher and lower end of the spectrum resulting in spurious values for M_max and M_min. We have shown the results of this analysis applied to Run 10 in Figure 27. To address (1) we have counted peaks partially by using a sigmoid function centered on the threshold to assign weights to the peaks. This means that peaks with intensities well below the threshold (<1%) have weighted closer to 0, and peaks well above the threshold (100x) are weighted effectively 1.0. Close to the weights assigned to the peaks move slowly between 1.0 (above the threshold) to 0.5 (at the threshold) then down to (0.0) well below the threshold. The mathematical form is: $w(i) = 1.0/(1 + \exp(-i + t))$,, where i is the intensity of the peak and t is the threshold. In place of counting the peaks (n_peaks) we sum the weights for the peaks.

For (2) instead of taking the maximum mass and the minimum mass we take the top 5% and bottom 5% m/z values and average them to replace M_max and M_min respectively. This means that the maximum mass is not just an outlier but instead the end of the distribution of observed masses. Similarly for the minimum mass. We found that the numerical value of the mass index has changed but the general trends have not.

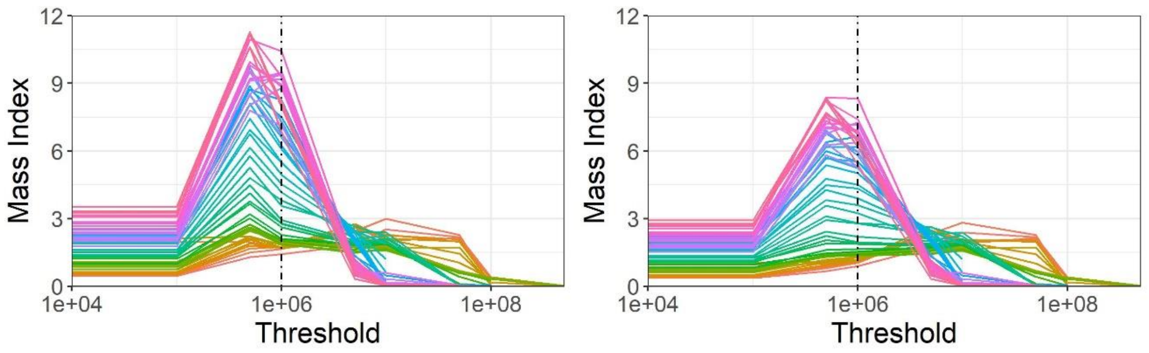

Supplementary Figure 26: Change in Mass Index based on noise filtering threshold. (Left) Here we show the Mass Index calculated for all the samples in Run 10 evaluated using different noise thresholds. Each line is one sample from Run 10. If the threshold is set too low, the mass index is dominated by

peaks from electrical noise. Which is removed around 10^5-10^6, resulting in the increase in the mass index (because the number of total peaks is greatly reduced by removing noise. Setting the threshold too high ~10^7, reduced the mass index by filtering true signal peaks. The different colours represent different samples while the black vertical dashed line indicates the threshold used during the experiments. (Right) the same analysis shown with the adaptive calculation in described in 5.4.1.

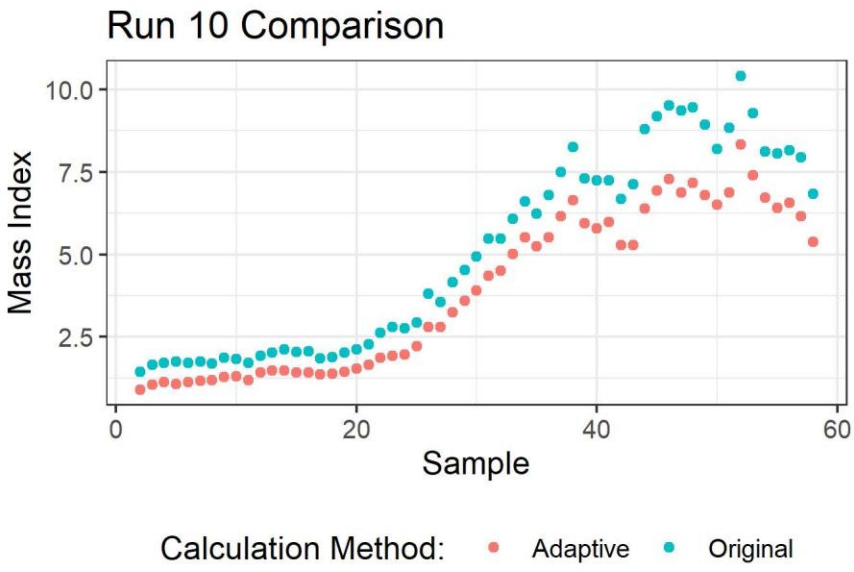

Supplementary Figure 27: Run 10 with adaptive and original mass index calculation

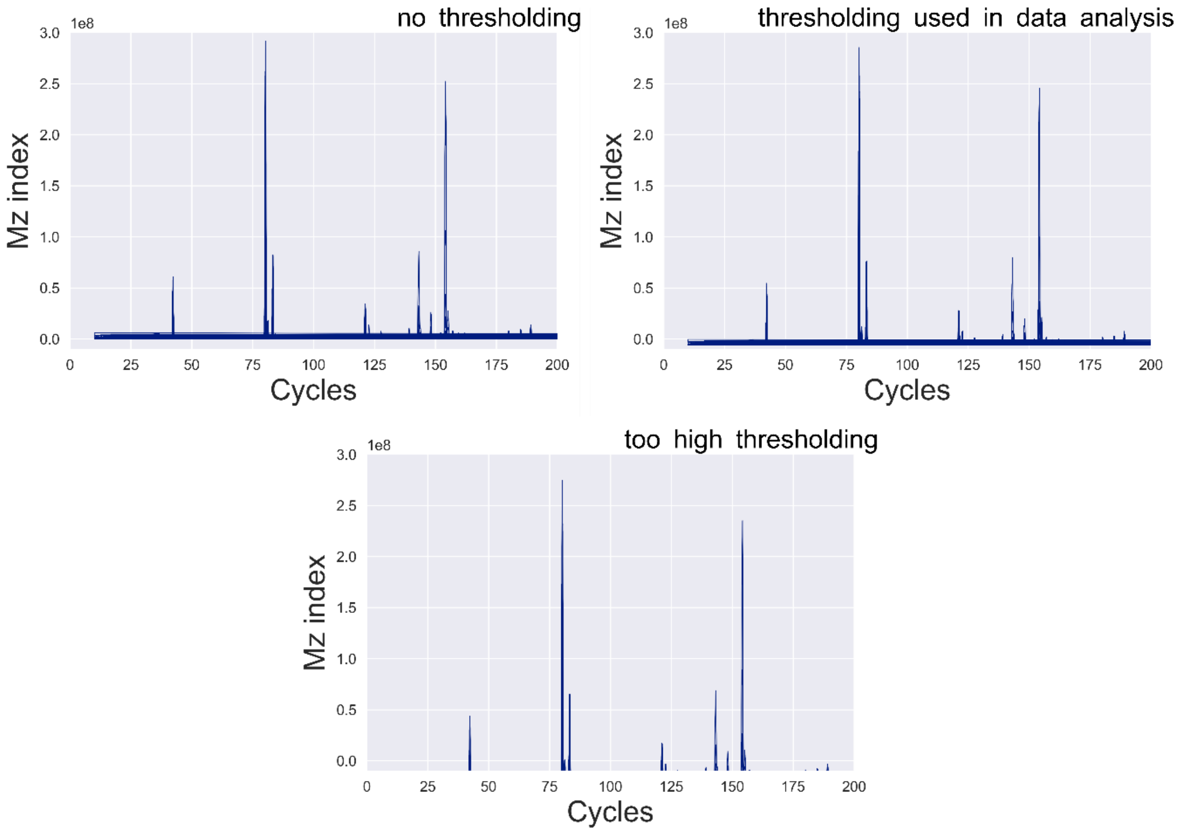

Supplementary Figure 28: Examples of thresholding tests for data analysis of offline samples. The top left shows no thresholding and the top right is the thresholding used in the data analysis. The

baseline is cleared up, while small peaks are still included. The bottom graph shows an example of too high thresholding, as baseline and small peaks are substantially cut off.

As these algorithms have been written post-data acquisition, thresholding was applied and adjusted directly to the experimental data. The best way for thresholding turned out to take the median of the intensities added to half of a standard deviation unit, see Supplementary Figure 29 and Supplementary Figure 28. This way of thresholding was chosen as it enables the code to capture every single peak of a spectrum, while filtering the lowest amount of small peaks over noise. This was important, as we wanted to take species into our calculation that are too low in abundance to be over a set threshold, but are different from starting material and noise. In the first part of the figure, all required libraries are imported. The function starts with reading the csv data into a data frame. In the second part of the function, the threshold of each intensity is calculated and added as a column to the dataframe. Dataframes are objects to store tabular data, specific for the data analysis toolkit Pandas, which can be used in Python. This thresholding strategy is used for every described code below.

```
import numpy as np
from numpy import array
from numpy import matrix
import pandas as pd
import matplotlib.pyplot as plt
%matplotlib inline

def get_df(df_file):
    #opens csv file and reads it in as a df
    my_df = pd.read_csv(df_file)
    list(my_df.columns.values)
    #set header right
    my_df.columns = ["rts", "mz", "int"]

    #adds another column to the dataframe with the intensity substracted by our set threshold
    #our threshold is the average threshold with the standard deviation divided by two added
    threshold = ((my_df.loc[:, "int"].median()) +  (my_df.loc[:, "int"].std()) / 2)
    thresh_df = my_df["int"] - threshold
    #add thresholding column
    my_df["threshold"] = thresh_df
    my_df[my_df < 0] = 0


    #this adds a column to the dataframe where we multiply mz mass with the thresholded intensity
    mz = my_df["mz"]
    t_int = my_df["threshold"]
    multi = mz * t_int
    my_df["multi"] = multi
    return(my_df)
```

Supplementary Figure 29: First part of the modified Mass Index code. The first block shows the libraries imported, the function shown here opens a csv file and adds a column with the calculated threshold to the dataframe. In the last part the threshold column is multiplied with the mass

The last part of Supplementary Figure 29 shows how the mass is set in relation with the intensity of the peak. Another column “multi” is added to the data frame in which the mass of each peak is multiplied with the thresholded intensity of each peak. Instead of using just the mass value in the Mass Index before, we are looking for the heaviest and lowest peak and are saving the corresponding multiplied value of this particular peak into two separate list. After that, the number of peaks is determined for the

whole spectrum and this number is stored for each retention time group (representing the spectra in the dataframe) in an additional list. With these three lists, it is possible to calculate the modified Mass Index, see Supplementary Figure 30.

```
def mz_index_calculation(my_df):
    #this calculates the actual mz index value
    list_max_number = max_number(my_df)
    list_min_number = min_number(my_df)
    peak_count = peak_counter(my_df)

    #first substract the minimum number from the maximum for every value in both lists
    a = matrix(list_max_number)
    b = matrix(list_min_number)
    sub = a - b
    #we convert the substraction list into an array that we can devide later
    sub_array = array(sub)
    #turns peak count into an array
    peak_array = array(peak_count)
    #divides the substracted min and max values through the number of peaks
    mz_list = np.divide(sub_array, peak_array)
    #takes the average of the total mz calculation
    mz_value = np.average(mz_list)
    return(mz_value)
```

Supplementary Figure 30: Function to calculate the modified Mass Index, using the python libraries numpy arrays and panda data frames

To compare both evaluation methods, the mass spec data from the inline-measured samples was rerun with the modified version of the Mass Index code (Supplementary Figure 30). The comparison is to be made carefully, as not just the relation between peaks and intensity is changed but on top of that, the thresholding. The values of the plotted data changed completely, so the comparison of these two data sets is limited to the observation of “data trends” and can be seen in Supplementary Figure 31.

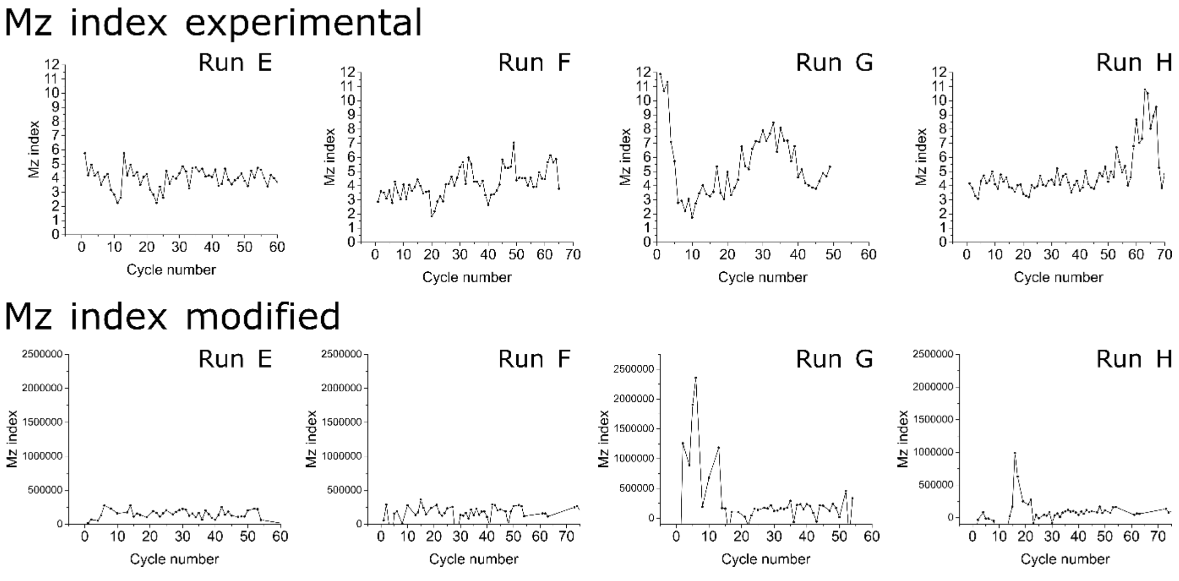

Supplementary Figure 31: Data evaluated with experimental Mass Index (mz index) and modified version, applied on same data

The plots presented in Supplementary Figure 31, do not show an immediate trend, which would be possible to observe. In general, the modified Mass Index seemed to have lowered the complexity or variation of specific cycles. In the experimental Mass Index comparison, all runs are in a similar scale, as when a similar scale is applied to the modified Mass Index, one run, run G stands out the most. This correlates with the experimental Mass Index, as the experimental Mass Index starts very high, until it drops around cycle 10. The modified Mass Index does has a rise in the beginning but the pattern of being high and dropping around cycle 10 can be observed in this case too. Run E and F are looking fairly similar in both calculations and run H seemed to be turned over its vertical axis as there is a rise visible at the end in the experimental Mass Index and with the modified version, a rise is possible to observe around cycle 20, which drops again afterwards. The algorithm would have clearly made different input choices than the experimental algorithm, but if this algorithm would make more sense than the mx index that was in use is questionable.

5.4.2 Weight by intensity index

An evaluation method based on the weight and the intensity of each peak was developed. In this calculation, the aim is to get a number z, which is the sum of all intensities multiplied by their mass value over each spectrum.

$$z = \sum I_p * \frac{m}{z}_p .$$

When this value is high, the sample has a higher amount of larger products, with a stronger signal.

As just multiplying every value would lead to very high numbers, the intensity is normalised before multiplied with the mass value (Supplementary Figure 32), similar to the multiplication step in the calculation for the modified Mass Index (Supplementary Figure 29).

```python
def normalise(my_df):
    #we are iterating through every retention time group
    unique_rts = sorted(list(set(my_df["rts"])))
    n_rt = len(unique_rts)
    #add another line into our df with normalised intensities ip, currently just with zeros stored
    my_df["normed_I"] = np.zeros(len(my_df["int"]))
    #want to get intensities for each spectra normalized by the total intensity for that spectra
    #(e.g. the total intensity for each rt should be = 1)
    for i in range(n_rt):
        #iterates through every retention time group
        rt_df = my_df[my_df["rts"]==unique_rts[i]]
        #sums up all intensities in a rt group
        T = sum(rt_df["int"])
        #divides the sum of all intensities T through each intensity in our df
        rt_df.loc[:,"normed_I"] = rt_df.loc[:,"int"]/T
        #adds or rt_df into our main df as our normed_I column
        my_df[my_df["rts"]==unique_rts[i]] = rt_df
    return(my_df)

def multiply(my_df):
    # add multiplied mz with threshold intensity
    mz = my_df["mz"]
    int_norm = my_df["normed_I"]

    multi_normed = mz * int_norm
    my_df["multi_normed"] = multi_normed
    return(my_df)

def sum_it_up(my_df):
    #sums the multi column and returns the multi sum
    total_sum = my_df["multi_normed"].sum()
    print(total_sum)
    return(total_sum)
```

Supplementary Figure 32: Functions for the calculation of the normalised mass over intensity value

In the last part of Supplementary Figure 32, the sum of all normed and multiplied values of the cycle is taken and saved to a list. The experimental inline Advion data is rerun with the new calculation and the data is presented in Supplementary Figure 33. In this figure, the weight by intensity value of run E to H is shown. As all of these runs have different input sets, there is not much to compare in between these runs but when the data is compared with the previous algorithms in Supplementary Figure 31, some differences are visible. As these are different calculations, the values on the y-axis on every plot are incomparable, therefore is the comparison limited to a description of the shape based on the cycle number.

Interestingly run E differs the most from the previous calculated Mass Index values. The weight by intensity values are compared to the other plots calculated with the same algorithm much higher, which leads to an elevation of the whole graph in the plot while the values are in the same data range as all other calculated values in data compared with the Mass Index in Supplementary Figure 31. Run F looks similar as in the modified Mass Index. While the experimental Mass Index has a clear rise between cycle 30 to 40 and again at approximately, cycle 50. The weight by intensity calculated values and the modified Mass Index values have several small peaks but no clear rise throughout the run. The weight by intensity run G is more similar to the experimental Mass Index calculation, showing a rise right in the

beginning, but the rise later in the experimental Mass Index calculation, visible in Supplementary Figure 31 is not observable in the values shown in Supplementary Figure 33.

Run H differs significant from the Mass Index calculations. There is no massive peak observable, but the values show a rise around cycle 30, which is not visible in the data previously calculated. An overlap with the experimental Mass Index is that another peak between cycle 60 and 70 is visible, which is a very distinct peak in the graph of the experimental Mass Index of run H shown in Supplementary Figure 31.

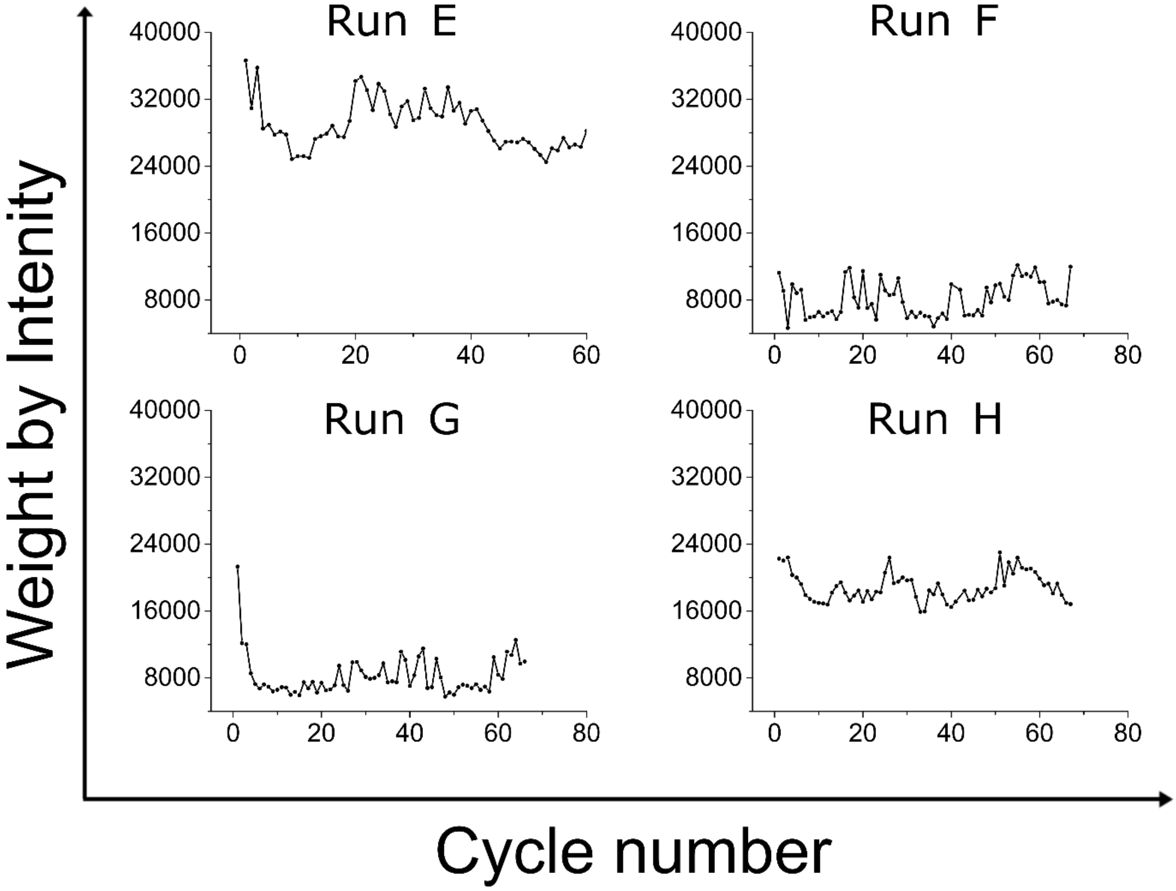

Supplementary Figure 33: Data evaluated with the weight by intensity calculation.

5.4.3 Information entropy value

In this approach, a code is developed to compare the cycles of a run based on their information entropy. Entropy is often defined as a value for the disorder of a system, but in this case, it is used to describe the information content in our system (*2*). The information entropy of a spectrum is defined as:

$$S = -\sum_{p} i_p * \ln(i_p).$$

Where $i_p = \frac{I_p}{T}$, and $T = \sum_p I_p$,i_p is the intensity of peak p normalized to the total intensity of the spectra. This leads to a value which will be lower when the sample has fewer, larger peaks and higher when the sample has many peaks of comparable size. The code starts in a similar manner to the previous ones by transferring the data into a pandas dataframe and setting the threshold for each intensity value. The intensity values are being normalised but this time not multiplied with the mass values. How the entropy value is calculated is shown in Supplementary Figure 34.

```
def calculate_entropy(my_df):
    #we are iterating through every retention time group
    unique_rts = sorted(list(set(my_df["rts"])))
    n_rt = len(unique_rts)
    #this adds a column to the dataframe that has the ln of ip stored
    my_df["log_ip"] = np.log(my_df["normed_I"])
    #this adds a column that has the ln of ip multiplied to ip stored
    my_df["entropy"] = my_df["log_ip"] * my_df["normed_I"]
    #create an empty list
    entropy = []
    #we are iterating through every retention time group again
    unique_rts = sorted(list(set(my_df["rts"])))
    n_rt = len(unique_rts)
    for i in range(n_rt):
        #iterates through every retention time group
        rt_df = my_df[my_df["rts"]==unique_rts[i]]
        #sums up the entropy for a spectra by summing it up for a retention time group and
        #stores it into our entropy list
        S = sum(rt_df["entropy"])
        entropy.append(S)
    #takes the average of the entropy of all spectras
    total = statistics.mean(entropy)
    print(total)
    return(total)
```

Supplementary Figure 34: Function to calculate the entropy value of Advion data

This function iterates through each retention time group and adds a column of the natural logarithm of each individual normed intensity to the data frame. This value is then multiplied with the value of the normed intensity. As the value desired is the entropy over a full spectrum, the code iterates through the data frame again, summing up all entropy values for each individual retention time group, resolving in the entropy value for each individual spectra. To generate a through the run comparable number, the average of the entropy of all spectra is taken and returned as entropy value for the individual cycle. The resulting data is shown in Supplementary Figure 35.

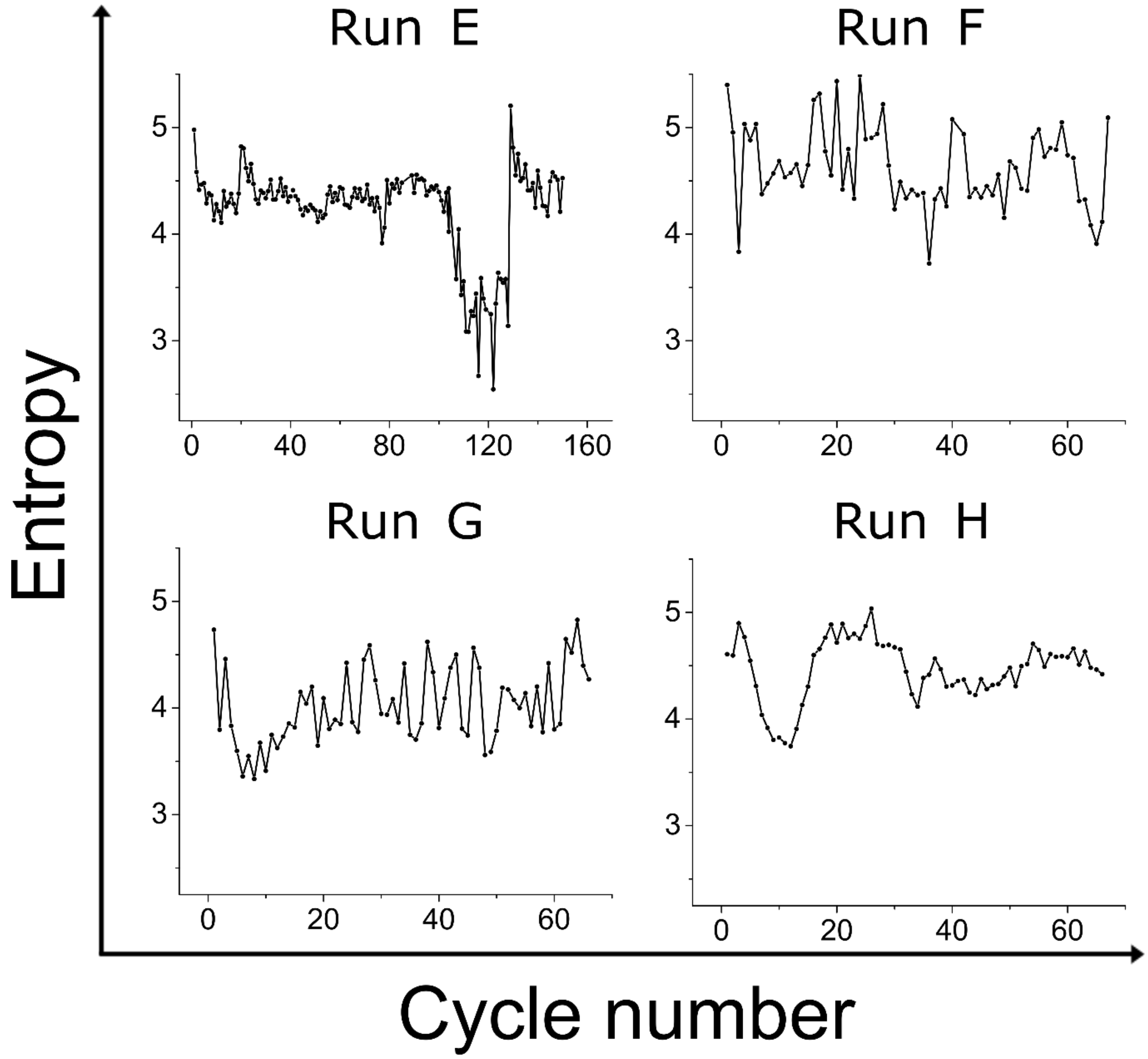

Supplementary Figure 35: Data evaluated with the information entropy measure

As before, the data will be compared with the Mass Index used in the experiment, as this was the code of which the input decisions have been based on. The graph for the entropy in run E in Supplementary Figure 35 is moderate throughout the first 100 cycles and declines steep after that until it rises again around cycle 130 to cycle 150. The graph has no similarities to the Mass Index calculates graph in Supplementary Figure 31 though.

Different to the entropy graph of run F that follows approximately a similar pattern than the graph of the Mass Index calculation but looks almost random. Both figures show a quite disorganised pattern but both plots rise between cycle 20 and 40. The run G plots differ heavily from each other. The Mass Index graph in Supplementary Figure 31 is high in the beginning, descends until cycle 10 and follows a curve after that. The information entropy graph in Supplementary Figure 35 of run G shows random distribution of points and the value is going rapidly up and down between cycles. Run H is different, as the entropy plot declines until cycle 10, rises after that until it reaches cycle 20 to cycle 55, where it

starts to follow another more moderate curve. The Mass Index plot of run H in Supplementary Figure 31 shows a moderate course until the index rises rapidly after cycle 60.

If we compare the algorithmically produced data of each run individually, we will compare the modified Mass Index, the weight by intensity and the information entropy as the same thresholding strategy was used in all 3 calculations. It turns out, the different algorithms give almost complimentary information about the individual runs. While run E has the highest weight by intensity value in comparison with the other 3 runs, which leads to the idea that this run has many product species of high mass value and high abundance, the information entropy of this run shows a drop around cycle 120. This means that in this range of cycles, rather than having a high amount of many product species we got a few very large peaks, which is plausible based on the weight by intensity value, as this value cannot distinguish between a high amount of peaks and a few intense peaks, in opposite to the information entropy value. On the same side, when considering the Mass Index, it suggests a number of larger peaks as if there would just be one dominant peak, this value would be high too. For run F, the weight by intensity index is relatively low throughout the run, this would lead to the idea, that there was a lower amount of species and the overall abundance of the signal was lower too. This explains the high information entropy, as it suggests many peaks of comparable size, which do not contradict the weight by intensity value. The Mass Index of run F is low, which shows that there is no dominant species of high mass, but rather a higher amount of peaks of lower abundance. The interpretation of run G appears more complex. The Mass Index value shows a high rise in the beginning of a run, suggesting an abundant dominant species in the beginning, which breaks down throughout the run. This can be further validated by the weight by intensity value, as this value shows a rise in the begin of the run. The information entropy, shows a drop in the same area, suggesting a fewer number of peaks but not such a clear trend as when calculated with the other two values. Run H shows a peak in the area between cycle 10 and 20, which suggests the build-up of a few species of higher mass, which break down into more species afterwards. This rise is not clear to read in the weight by intensity value as this value shows a small rise at that point but a more dramatic rise later in the run, in which the mz value does not show a rise. On the other hand, the information entropy shows a drop, suggesting that there are rather few larger species, like the Mass Index suggested. It is interesting to see that observations based on the different algorithms can, if handled carefully, build on each other. On the other it is important to state, that the algorithms alone are just able to give ideas of the product distribution in a sample and if a cycle contains dominant product species. For more information about the exact chemical composition and the reactions, which did occur in the system, a more extensive analysis is necessary.

6 Supplementary References

1. D. Doran, Y. M. Abul-Haija, L. Cronin, Emergence of Function and Selection from Recursively Programmed Polymerisation Reactions in Mineral Environments. *Angewandte Chemie International Edition* **58**, 11253-11256 (2019).
2. S. Vajda, C. E. Shannon, W. Weaver, The Mathematical Theory of Communication. *The Mathematical Gazette* **34**, (1950).
