# Supplementary material for: A robotic prebiotic chemist probes long term reactions of complexifying mixtures
Source: Nat Commun. 2021 Jun 10;12:3547. doi: 10.1038/s41467-021-23828-z (PMC8192940; doi:10.1038/s41467-021-23828-z)
Supplement: Supplementary file 3 — Description of Additional Supplementary Files [file 41467_2021_23828_MOESM3_ESM.docx]

Description of Additional Supplementary Files

Title: Supplementary Data 1

Description: This folder contains all the information and code required to recreate the figures of the manuscript.
