# Supplementary material for: A robotic prebiotic chemist probes long term reactions of complexifying mixtures
Source: Nat Commun. 2021 Jun 10;12:3547. doi: 10.1038/s41467-021-23828-z (PMC8192940; doi:10.1038/s41467-021-23828-z)
Supplement: Supplementary file 4 — Supplementary Dataset 1 [file 41467_2021_23828_MOESM4_ESM.zip › Data/3F8160F0.tmp]

**This folder contains all the information and code required to recreate the figures from ‘A robotic chemist probes long term reactions of prebiotic mixtures’ by Silke Asche, Geoffrey J. T. Cooper, Graham Keenan, Cole Mathis and Leroy Cronin (2020)**

Unless otherwise stated the figures were generated using OriginPro 2016 on Windows 10. The data provided is provided as Origin Files.

**Figure 1**

The chemical structures in Figure 1A have been drawn using ChemDraw Prime and have been provided in Molecules.cdx.

Figure 1B was drawn using the package `graph_tool` (<https://graph-tool.skewed.de/>) which is a python 3 wrapper for a C++ library. The Jupyter notebook in ‘Figure 1/Draw_with_GraphTools-clean.ipynb’ can be used to generate the figure. The reactions predicted from Molecular Transformer are in `Figure 1/data/` saved as .csv files with separate files for the 2 and 3 component reactions. Notebooks to generate the graph from the reaction data are in the same folder. This notebooks works with Python 3.7 and Jupyterlab v1.x

**Figure 4**

The “data_example” folder contains the Advion file formats (.cdf and .datx) as well as a converted csv file of exemplary experimental data. Full dataset available upon request.

The mass index numbers listed in the corresponding csv files have been generated by “advion_spectrum.py” which takes the Advion cdf file and returns the mass index number of each specific cycle. The “decision_maker.py” takes these numbers and calculates the slope between cycles to decide if the experiment must be changed. “decision_maker.py” depends on the custom built robotics platform described in the SI. Both scripts use Python 3.8. An example of how to use the advion_spectrum.py script to calculate the mass index is given here

The “Advion_species_count” code takes the converted Advion csv-files and counts the number of unique ions in the cycles of an experiment.

The plotted results with the corresponding data can be found in “Fig4_plotting.opj”.

**Figure 5**

The data_example folder shows the mzML files of the original Orbitrap files, which have been converted with msConvert. The mzML files have been then converted to json files using proprietary software. Files of both data types are provided.

The “Orbi_reproducibility.py” code takes the json input files, thresholds the data and creates a list of all unique ions, which is stored as “Output_orbi_species_count.xlsx”.

The compared Orbitrap data is plotted in “Orbi_repro_filtered_v3.opj” and the comparison of the Advion data as mass index is plotted in “Comparison_Run_10_11.opj”.

**Figure 6**

The data_example folder shows csv files of the Advion data, copied out of the Advion Data Express program.

The data was thresholded and the highest mass was selected for each cycle.

The data is plotted in “Highest_mass_in_cycles_Advion.opj”.
