# Supplementary material for: A robotic prebiotic chemist probes long term reactions of complexifying mixtures
Source: Nat Commun. 2021 Jun 10;12:3547. doi: 10.1038/s41467-021-23828-z (PMC8192940; doi:10.1038/s41467-021-23828-z)
Supplement: Supplementary file 4 — Supplementary Dataset 1 [file 41467_2021_23828_MOESM4_ESM.zip › Data/Figure 4-Revision/Mass_species_count_SIA6-74/Species count.rtf]

import csv
import os
import filetools
import inspect

def make_list_csv(data_folder):
    
    csvs = [
        os.path.join(data_folder, r_file)
        for r_file in os.listdir(data_folder)
        if r_file.endswith('csv')]
    return csvs

def main(data_folder):
    list_csv = make_list_csv(data_folder)
    
    
    
def get_df(csv_file):
    #opens specfific csv file and reads it in as a df
    my_df = pd.read_csv(csv_file)
    list(my_df.columns.values)
    #set header right
    my_df.columns = ["rts", "mz", "int"]
    #adds another column to the dataframe with the intensity substracted by our set threshold
    #our threshold is the average threshold with the standard deviation divided by two added
    threshold = ((my_df.loc[:, "int"].median()) +  (my_df.loc[:, "int"].std()) / 2) 
    thresh_df = my_df["int"] - threshold
    #add thresholding column
    my_df["threshold"] = thresh_df
    #everything below 0 is 0
    my_df[my_df < 0] = 0
    new_df = my_df[my_df["threshold"] != 0]
    
    #this adds a column to the dataframe where we multiply mz mass with the thresholded intensity
    #it does not matter if we count mz or multi as all 0 int will make zero multi which will not be counted
    # a problem is though if m/z values come up multiple times
    
    return(new_df)
    
    
def get_count(new_df):
    list_mass = []
    list_mass = new_df['mz'].values.tolist()
    newer_list = []
    #get rid of duplicates
    for value in list_mass:
        if value in newer_list:
            pass
        else: 
            newer_list.append(value)
    return len(newer_list) 
    
    
def main(data_folder):
    list_csv = make_list_csv(data_folder)
    for csv_file in list_csv:
        new_df = get_df(csv_file)
        print(csv_file)
        count = get_count(new_df)
        print(count)
        
       # mz_value = mz_index_calculation(my_df)
     #   out_rows.append([csv_file, mz_value])
   # save_to_csv("OutputTraditional.csv", out_rows)
   # print("calculation finished")
    
main(data_folder="C:/Users/Group/Silke code/advion")

#if __name__ =="__main__":
    #main()
