# Supplementary material for: A robotic prebiotic chemist probes long term reactions of complexifying mixtures
Source: Nat Commun. 2021 Jun 10;12:3547. doi: 10.1038/s41467-021-23828-z (PMC8192940; doi:10.1038/s41467-021-23828-z)
Supplement: Supplementary file 4 — Supplementary Dataset 1 [file 41467_2021_23828_MOESM4_ESM.zip › Data/readme.docx]

**This folder contains all the information and code required to recreate the figures from ‘A robotic chemist probes long term reactions of prebiotic mixtures’ by**  **Silke Asche, Geoffrey J. T. Cooper, Graham Keenan, Cole Mathis1 and Leroy Cronin (2020)**

**Figure 1**

COLE

**Figure 4**

The “data_example” folder contains the Advion file formats (cdf and datx) as well as a converted csv file of exemplary experimental data.

The mass index numbers listed in the corresponding csv files have been generated by “advion_spectrum.py” which takes the Advion cdf file and returns the mass index number of each specific cycle. The “decision_maker.py” takes these numbers and calculates the slope between cycles to decide if the experiment must be changed.

The “Advion_species_count” code takes the converted Advion csv-files and counts the number of unique ions in the cycles of an experiment.

The plotted results with the corresponding data can be found in “Fig4_plotting.opj”.

**Figure 5**

The data_example folder shows the mzML files of the original Orbitrap files, which have been converted with msConvert. The mzML files have been then converted to json files, files of both data types are stored.

The “Orbi_reproducibility.py” code takes the json input files, thresholds the data and creates a list of all unique ions, which is stored as “Output_orbi_species_count.xlsx”.

The compared Orbitrap data is plotted in “Orbi_repro_filtered_v3.opj” and the comparison of the Advion data as mass index is plotted in “Comparison_Run_10_11.opj”.

**Figure 6**

The data_example folder shows csv files of the Advion data, copied out of the Advion Data Express program.

The data was thresholded and the highest mass was selected for each cycle.

The data is plotted in “Highest_mass_in_cycles_Advion,opj”.
